# Applying molecular and genetic methods to trees and their fungal communities

**DOI:** 10.1007/s00253-023-12480-w

**Published:** 2023-03-29

**Authors:** Markus Müller, Ursula Kües, Katharina B. Budde, Oliver Gailing

**Affiliations:** 1 Forest Genetics and Forest Tree Breeding, Faculty for Forest Sciences and Forest Ecology, University of Goettingen, Büsgenweg 2, 37077 Göttingen, Germany; 2 Center for Integrated Breeding Research (CiBreed), University of Goettingen, 37073 Göttingen, Germany; 3 Molecular Wood Biotechnology and Technical Mycology, Faculty for Forest Sciences and Forest Ecology, University of Goettingen, Büsgenweg 2, 37077 Göttingen, Germany; 4 Center for Molecular Biosciences (GZMB), Georg-August-University Göttingen, 37077 Göttingen, Germany; 5 Center of Sustainable Land Use (CBL), Georg-August-University Göttingen, 37077 Göttingen, Germany

**Keywords:** Holobiont, Conservation, Interaction, Genome, Sequencing

## Abstract

**Abstract:**

Forests provide invaluable economic, ecological, and social services. At the same time, they are exposed to several threats, such as fragmentation, changing climatic conditions, or increasingly destructive pests and pathogens. Trees, the inherent species of forests, cannot be viewed as isolated organisms. Manifold (micro)organisms are associated with trees playing a pivotal role in forest ecosystems. Of these organisms, fungi may have the greatest impact on the life of trees. A multitude of molecular and genetic methods are now available to investigate tree species and their associated organisms. Due to their smaller genome sizes compared to tree species, whole genomes of different fungi are routinely compared. Such studies have only recently started in forest tree species. Here, we summarize the application of molecular and genetic methods in forest conservation genetics, tree breeding, and association genetics as well as for the investigation of fungal communities and their interrelated ecological functions. These techniques provide valuable insights into the molecular basis of adaptive traits, the impacts of forest management, and changing environmental conditions on tree species and fungal communities and can enhance tree-breeding cycles due to reduced time for field testing. It becomes clear that there are multifaceted interactions among microbial species as well as between these organisms and trees. We demonstrate the versatility of the different approaches based on case studies on trees and fungi.

**Key points:**

*• Current knowledge of genetic methods applied to forest trees and associated fungi.*

*• Genomic methods are essential in conservation, breeding, management, and research.*

*• Important role of phytobiomes for trees and their ecosystems.*

## Introduction

Forests cover approximately 31% of the global land area and provide invaluable economic, ecological, and social services, such as the provision of food, timber, income, habitat for a variety of species of all organismal kingdoms, carbon sequestration, nutrient cycling or the prevention of soil erosion (FAO and UNEP 2020). In the face of global change, forests are exposed to several threats, such as fragmentation and deforestation, changing climatic conditions, increasing demand for forest products, and increasingly destructive autochthonous and alien-introduced pests and pathogens. Research on the adaptation of forests to these challenges needs a multifaceted approach, in which genomic analyses play an important role (Plomion et al. 2016). A better understanding of tree genetics and genomics could help to increase the success of conservation initiatives, inform about the capacity of tree populations to adapt to changing climatic conditions, and facilitate new breeding methods that accelerate breeding cycles and improve the accuracy of breeding values (Grattapaglia et al. 2018; Isabel et al. 2020). Despite the importance of trees, knowledge of their genetic system and constitution is still much more restricted compared to e.g. agricultural plants. Trees are mostly undomesticated, non-model species with a long lifespan and a wide range of genome sizes for which genomic resources have only recently been developed. The first tree genome, of black cottonwood (*Populus trichocarpa*), was published in 2006 (Tuskan et al. 2006). Since then, the rapid development of high-throughput sequencing (HTS) and of new approaches to analyze the genetic properties of species has also led to a better understanding of tree genetics. New genetic methods are used in several forest genetic fields, such as conservation genetics, tree breeding, phylogenetics, or the analysis of tree-associated microbial communities (Table 1).

**Table 1:** Overview of different genetic methods and their advantages and disadvantages

Method	Applications	Advantages	Disadvantages	References
Classical genetic markers and methods	Measurement of genetic diversity/differentiation, analysis of the mating system, barcoding, association studies, analysis of hybridization	Cheap, applicable in non-model species, due to low amount of generated data comparatively easy to analyze	Only a fraction of the genome is analyzed	Eriksson and Ekberg (2001); Finkeldey et al. (2020)
Whole genome sequencing and re-sequencing	Analysis of copy number variation (CNV), analysis of genome evolution, establishment of reference genomes	Analyses of full genomes	Costly, large computational resources needed	Plomion et al. (2016); Holliday et al. (2019)
Genome complexity reduction	Investigation of population structure, single nucleotide polymorphism (SNP) identification, phylogenetics, analysis of epigenetic variation	Cost-efficient (e.g., genotyping by sequencing (GBS)), targeting of specific genomic regions possible (depending on the method, e.g., sequence capture), obtaining genome-wide data, applicable in non-model species	Some methods costly (e.g., sequence capture), no targeting of specific genome regions (depending on the method, e.g., GBS)	Gasc et al. (2016); Parchman et al. (2018); Holliday et al. (2019); Hipp et al. (2020)
RNA-seq	Analysis of transcriptomes, analysis of gene expression in response to abiotic/biotic stressors, identification of gene expression networks	Applicable in non-model species, cost-effective	Potentially missing low-abundance transcripts	Weber (2015); Harper et al. (2016); Holliday et al. (2019); Wu et al. (2019); Zinkgraf et al. (2020)

**Fig. 1:**
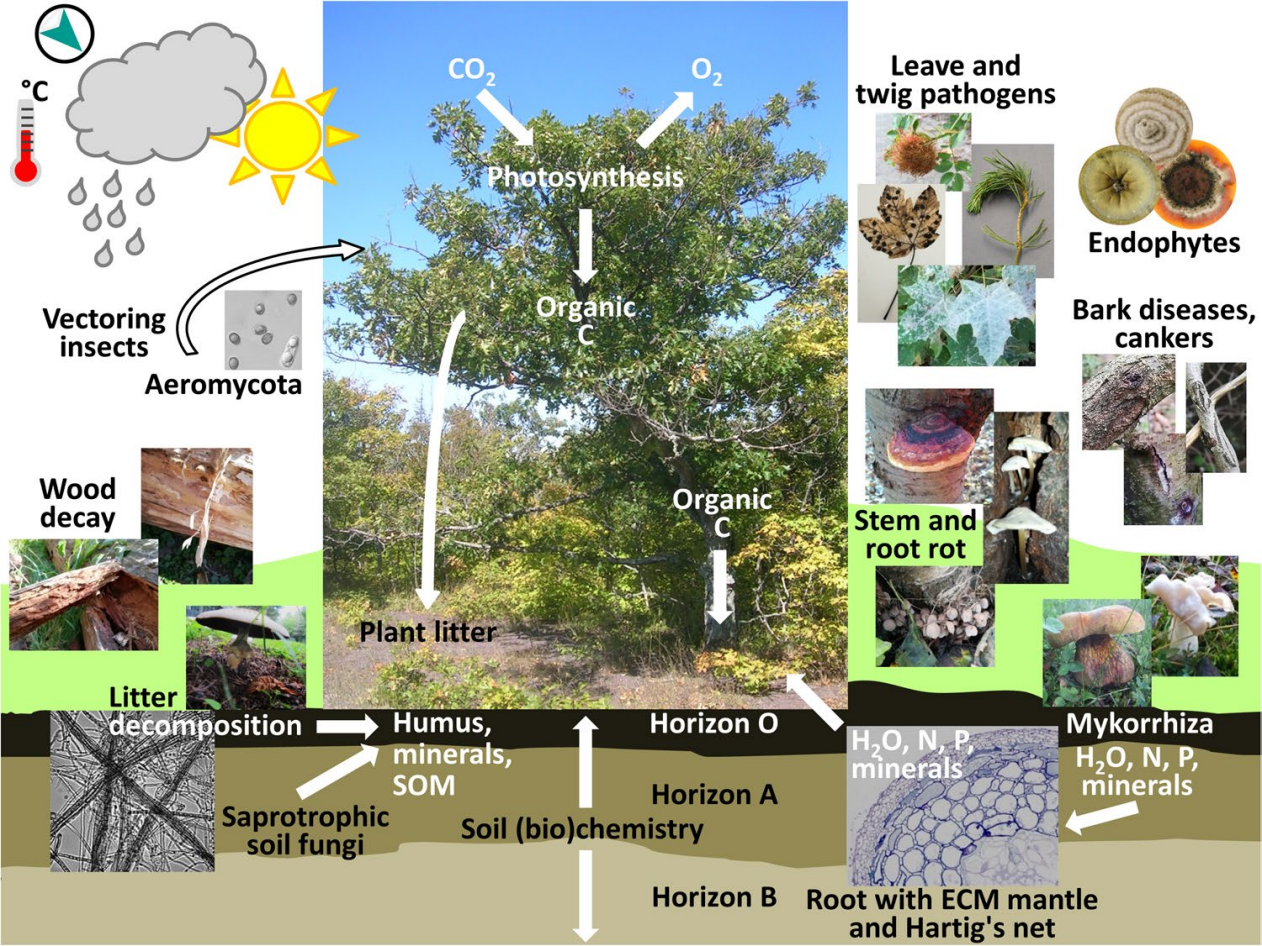
**A tree phytobiome consists of the tree, its environment, and all organisms living in, on and around the plant.** Growth of a photosynthetic tree is directly and indirectly influenced by abiotic climatic conditions (rain, light, temperature, wind), by abiotic soil chemistry in its different horizons (O: organic horizon; A: surface horizon; B: subsoil) and by biological interactions with multiple other organisms (bacteria, fungi, animals, possibly other plants) and possibly viromes that may affect the quality of their growth substrate by participating in chemical turnovers and influence the tree performance via the growth substrate and in manifold other ways. The term phytobiome overlaps with the ecological concept of the holobiont as a functional entity of the plant and its associated communities of microbes, i.e. the phytomicrobiome, and viruses that will co-evolve with the tree under adaptations to changes in the environment. The species of the complex phytomicrobiome influence each other in abundance and composition and thereby also the tree, while the tree in turn modifies the abundance and composition of associated microbial species by secreting biochemical compounds (Lyu et al. 2021). Exemplified in the figure are the main roles in tree growth exerted by fungi: saprotrophs decay wood and other plant litter for mineralization and humus formation; mycorrhizal fungi help uptake limiting nutrients, such as N and P, minerals and water in return of organic carbon resulting from the photosynthetic activity of the tree; pathogens can occur on any plant organ and harm these by removal of nutrients and destruction; endophytes live seemingly neutral within plant tissues without obvious negative or positive effects on the host, but may e.g. protect the tree against harmful microbial intruders or change in lifestyles under other environmental conditions. Further, fungi of functional significance for the holobiont might occur at low abundance as epiphytes in the phyllosphere as a yet underexploited habitat. Such fungi may arrive at their places as part of aeromycota and may act in defeating pathogens present throughout the community of epiphytes, or as saprotrophs in preferred positions awaiting e.g. leaf and needle fall for rapid substrate colonization and decomposition (Bashir et al. 2022; Lindow and Brandl 2003; Zhu et al. 2021). Note that fungal organisms and the tree (northern red oak, *Quercus rubra*) shown in the figure were randomly chosen for demonstrations from a selection of photos available and do not necessarily occur together in a natural phytobiome

Trees cannot be considered as isolated organisms in environments. Accordingly, the phytobiome encompasses the plant, its environment, and all organisms living in, on, and around the plant. Environments are not static and trees are thus confronted manifold with changing biotic and abiotic influences (e.g., Adnan et al. 2022; Fortier et al. 2019; Frei et al. 2018; Teshome et al. 2020; Fig. 1). In an ecological and evolutionary context, the plant holobiont as functional entity comprises the plant and its associated microbes and also viruses that affect host growth and survival (Fig. 1), with poplars being used as holobiont models for trees (Cregger et al. 2021). Major environmental players in plant fitness are the diverse microbiomes (microbial communities) that associate e.g. with the rhizospheres (narrow soil regions around roots) or the phyllospheres (aboveground surfaces) of the trees, that live biotrophically or necrotrophically within distinct plant tissues, or that connect to plant litter and to wood degradation and will influence soil properties and nutrition in the stands of trees (Adnan et al. 2022; Langer et al. 2021; Nilsson et al. 2019; Terhonen et al. 2019; Fig. 1). Tree-associated microorganisms may largely consist of bacteria and fungi, and there can be archaea and various single-celled eukaryotic protists. Among them, fungi are likely to have the greatest impact on the life of trees (Baldrian 2017; Prescott and Grayston 2013; Zanne et al. 2020).

**Fig. 2:**
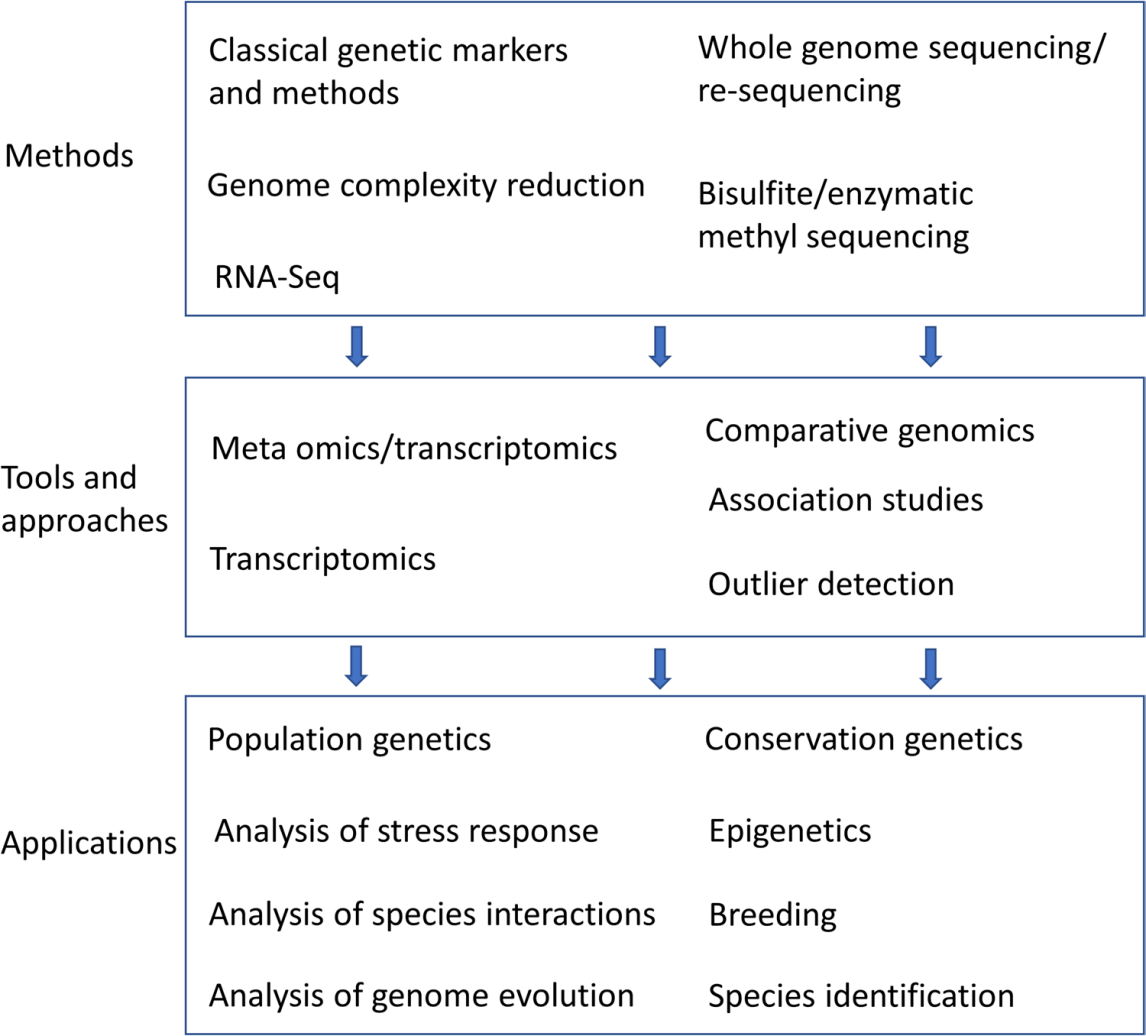
Links between genetic methods, tools/approaches, and applications discussed in this review. For details on the methods and tools see also Table 1 and Box 1. Methods generate genetic data, which are processed and analyzed with different approaches/tools, and can be used for different applications

The variety of molecular methods and the rapid development of new bioinformatic tools make it difficult for researchers to keep up-to-date and to choose appropriate approaches for a given study (Balkenhol et al. 2019). Therefore, studies investigating the suitability and potential limitations of different genetic methods are needed, as well as reviews summarizing the current understanding of the topic. Recently, excellent reviews have been published on genomic approaches applied in forest genetic and fungal research as well as the analysis of genetic and genomic data to investigate the evolutionary history and adaptive genetic patterns of tree species or taxonomic profiles of fungal communities and their functional and ecological attributes (e.g., Adnan et al. 2022; Genre et al. 2020; Holliday et al. 2019; Isabel et al. 2020; Lind et al. 2018; Nilsson et al. 2019; Plomion et al. 2016). Here, we focus on summarizing the application of different molecular and genetic methods to forest tree species and their associated fungal communities (i.e., their mycobiomes). We first give an overview of different methods mainly focusing on examples of their use in forest tree species. We start with classical genetic methods, which still play an important role in forest genetics research, followed by a description of applications based on whole genome and transcriptome data (Box 1). Subsequently, we describe how these methods can be (complementarily) used in forest conservation genetics, tree breeding, and association genetics (Fig. 2). Finally, we present how genetic and genomic approaches are used to investigate associated fungal communities and their influences on tree growth and ecology. Given the extensive ongoing research in the field of mycology, this overview here is mainly limited to fungi with life styles being in direct contact with tree hosts. The immense input of saprotrophs as another central part of the complete forest phytobiome ecology cannot be overlooked, which is why additional aspects of plant litter and wood decay are differentiated in a complementary article (Kües et al. in prep.).

**Box 1** Short descriptions of the genetic markers and methods discussed in this review

**Table:** 

**Isozymes:** different molecular forms of an enzyme with equal or similar catalytic function
**Simple Sequence Repeats (SSRs) or microsatellites:** short repeated DNA motifs typically of 1 to 6 bp length located in all regions of eukaryotic genomes
**Random Amplified Polymorphic DNAs (RAPDs):** a method, in which DNA is PCR-amplified using single primers with arbitrary nucleotide sequences
**Amplified Fragment Length Polymorphisms (AFLPs):** PCR-based method where selected restriction fragments are amplified with primer pairs complementary to synthetic oligonucleotides adapters ligated to the ends of the DNA fragments
**PCR-Restriction Fragment Length Polymorphisms (RFLPs):** specific DNA regions are amplified with primers and then cut with restriction enzymes
**Single Nucleotide Polymorphisms (SNPs):** SNPs are single sites (base pair positions) with different alleles present in a population
**DNA barcoding:** DNA barcodes are short taxonomically informative DNA regions that can be amplified and sequenced reliably across a wide taxonomic range of plants using primers in conserved regions of the genome
**Whole genome sequencing:** determination of the order of nucleic acids in the entire genome of an organism
**Genome complexity reduction:** methods used to generate reduced representation sequencing libraries. Genomic DNA can be digested using restriction enzymes or target DNA fragments are captured with synthetic baits
**RNA Sequencing (RNA-seq):** a method used to determine genome-wide gene expression. Dual RNA-seq describes the simultaneous transcriptomic analysis of different species based on the same sample (e.g., a mixed sample of a pathogen and its host)

## Classical genetic markers and methods

### Isozymes

Isozymes, different molecular forms of an enzyme with equal or similar catalytic function, have frequently been used as molecular markers for population genetic analyses in forest trees since the 1960s (Eriksson and Ekberg 2001). The use of a limited core set of isozyme loci allowed for comparative analyses of genetic variation and differentiation across plant species and revealed high levels of within-population genetic diversity in outcrossing and long-lived forest trees (Hamrick et al. 1992). Isozyme gene loci are codominant markers, distinguishing homo –and heterozygotes, which can be developed and applied across species at comparatively low costs (Holliday et al. 2019). However, only a few enzymes in a genome can be visualized by histochemical staining (seldom more than 25) so that the representation of the total genome is quite limited (Eriksson and Ekberg 2001). The low numbers of available markers and restrictions with respect to the detection of DNA-sequence and amino acid level substitutions led to the progressive replacement of isozymes by PCR-based DNA markers.

### Simple sequence repeats

Simple sequence repeats (SSRs) or microsatellites are regions of DNA with short segments of tandem repeats usually of 1–6 base pair length. They occur in all regions of eukaryotic nuclear genomes, most frequently in non-coding DNA (Weising et al. 2005). SSRs in non-coding regions (nuclear SSRs, nSSRs) are highly variable within populations making them useful for the identification of individuals or clones. The codominant mode of inheritance and high variability and allelic richness of nSSRs are determining factors for their wide use in gene flow and mating system analyses, and genetic diversity assessment also in tree species and populations with overall low genetic variation (Finkeldey et al. 2020). They are species-specific markers, often showing a low transferability across related taxa, especially when they are located in non-coding variable genomic regions. nSSRs have been developed for a large number of tree species, for example by developing and sequencing SSR-enriched libraries (Fischer and Bachmann 1998; Pandey et al. 2004) or by low-coverage genome sequencing (Staton et al. 2015). While predominantly occurring in intergenic regions, SSRs are also found in expressed genes (expressed sequence tags, ESTs), mostly in 5’ and 3’ untranslated regions, but also in coding regions (Ellis and Burke 2007). Especially trinucleotide repeats are found in coding regions since variation in repeat numbers does not disrupt the reading frame but can be associated with protein function and phenotypic traits. For example, an allele of a poly(Q) repeat (glutamine tract) was associated with growth cessation in *Populus tremula* (Ma et al. 2010). Variation in a trinucleotide SSR encoding for a glutamine tract in the coding region of a *CONSTANS*-like gene showed high interspecific differentiation as a signature of strong divergent selection between neighboring populations of two hybridizing oak species, *Quercus rubra* and *Quercus ellipsoidalis*, with different adaptations to drought (Lind-Riehl et al. 2014). Transcriptome libraries have been developed for an increasing number of tree species and can be used as a resource for the development of EST-SSRs (e.g., Breidenbach et al. 2020; Durand et al. 2010). In comparison to nSSRs, they show a higher transferability across related species, especially if primers are developed in conserved genomic regions (e.g., in coding regions of a gene) (Ellis and Burke 2007). Finally, SSRs are also present in organelle (chloroplast (cp) and mitochondrial (mt)) genomes (Weising and Gardner 1999; Weising et al. 2005). cpSSRs are frequently used in phylogeographic studies, especially in angiosperms with maternally inherited cpDNA (Finkeldey and Gailing 2013; Ndiade-Bourobou et al. 2020; Pettenkofer et al. 2019). In summary, also in the era of genomics, SSR markers still have a wide range of applications in tree population genetics.

### Random amplified polymorphic DNAs

Williams et al. (1990) introduced random amplified polymorphic DNAs (RAPDs), a technique based on random PCR-amplification of DNA using only single primers of the arbitrary nucleotide sequence. RAPDs have been used for population genetic studies in several species. In trees for instance, they were used to infer genetic variation and differentiation in *Sorbus torminalis*, a forest tree species with scattered distribution in Europe (Belletti et al. 2008), or to investigate genetic drift in small populations of *Taxus baccata* in Switzerland (Hilfiker et al. 2004). RAPDs were also used on its organismal companions, for instance to infer hybridization between two subspecies of *Ophiostoma novo-ulmi*, the ascomycetous pathogens that cause the Dutch elm disease (Brasier and Kirk 2010). RAPDs are inexpensive markers, which can be applied in uncharacterized genomes, but they also have some disadvantages, such as being dominant markers and showing issues of repeatability (Holliday et al. 2019).

### Amplified fragment length polymorphisms

The amplified fragment length polymorphism (AFLP) technique is a PCR-based method where selected restriction fragments are amplified with primer pairs complementary to synthetic oligonucleotides (Vos et al. 1995). A relatively large number of reproducible PCR fragments is generated in a single reaction which can be visualized and scored after electrophoretic separation on highly resolving polyacrylamide (PAA) gels or on a capillary sequencer. AFLPs are dominant (presence/absence) markers and their location in the genome is unknown. AFLP fragments can be gel-extracted, cloned, and sequenced to develop sequence characterized amplified region (SCAR) markers, but the method is technically demanding and time-consuming (Gailing and Bachmann 2003; Nuroniah et al. 2010, 2017). AFLPs have been widely used in genetic diversity assessments and genetic mapping in plants, including forest trees (Cao et al. 2009; Gailing et al. 2008, 2013; Meudt and Clarke 2007; Wang et al. 2014; Wehenkel et al. 2020). They are still valuable as a relatively low-cost genome-wide marker method for species for which no genomic or specific marker resources are available (e.g., in tropical tree families with high species diversity). However, AFLPs are more and more replaced by next-generation sequencing methods, such as restriction site-associated DNA sequencing (RAD-seq), which produces genome-wide single nucleotide polymorphism (SNP) markers (Kirschner et al. 2021).

### PCR-restriction fragment length polymorphisms

PCR-restriction fragment length polymorphisms (PCR-RFLPs) have been used frequently for the characterization of sequence variation in uniparentally inherited chloroplast DNA of plants including forest trees. Specific intergenic regions for example between the chloroplast transfer RNA genes, such as *trnL*, *trnL-trnF* (Taberlet et al. 1991), *trnD-trnT*, and *trnC-trnD* (Demesure et al. 1995) are amplified with conserved-gene-anchored primers (“universal primers”) and then cut with restriction enzymes. The PCR–RFLP technique is a cost-effective method to characterize chloroplast haplotype distribution across species ranges and has been used to reconstruct postglacial recolonization routes of major forest tree species in Europe and North America (Heuertz et al. 2004; Laricchia et al. 2015; Palme et al. 2003; Petit et al. 2002a, 2002b, 2003). Due to the pronounced phylogeographic structure generally observed at uniparentally inherited cpDNA markers, PCR–RFLP of cpDNA can be used to pinpoint the geographic origin of forest reproductive material or wood products (Jiao et al. 2019; Nuroniah et al. 2017; Rachmayanti et al. 2009).

### Single nucleotide polymorphisms in candidate genes

Single nucleotide polymorphisms (SNPs) are single sites (base pair positions) with different alleles present in a population. They are the main source of genetic variation in plant and animal genomes (Holliday et al. 2019) and have numerous applications in population and association genetics (Gibson and Muse 2004). SNPs in candidate genes for adaptive trait variation can be extracted from low coverage genome and transcriptome sequencing. SNP genotyping assays can be designed to analyze a few (ca. 20 to 200) SNPs in selected candidate genes or up to 700,000 SNPs in model tree species for which whole genome sequences and transcriptomes are available (Holliday et al. 2019).

### DNA barcoding

DNA barcodes are short taxonomically informative DNA regions that can be amplified and sequenced reliably across a wide taxonomic range of plants using primers in conserved (coding) regions of the genome. A standard set of barcoding regions has been used for the taxonomic identification of flowering plants, the chloroplast coding genes *rbcL* and *matK*, intergenic or intron chloroplast regions *trnL*, *trnH-psbA* and the internal transcribed spacer (ITS) region of ribosomal DNA (Hollingsworth et al. 2011; Kress et al. 2005, 2009). The Barcoding of Life Data System (BOLD) has been established as a reference database of DNA sequences linked to herbarium vouchers (Hollingsworth et al. 2009). The lack of sequences for many tropical species still limits the application of DNA barcoding for sample identification to the species level, while genus-level identification is often achieved (Moura et al. 2019). Also, the resolution of barcoding sequences is low for some taxonomic groups (e.g., species of the genus *Shorea* of the important tropical tree family *Dipterocarpaceae*) requiring genome-wide sequencing methods to better resolve species relationships (Heckenhauer et al. 2018, 2019).

## Whole genome sequencing and re-sequencing

Whole genome sequencing refers to the determination of the order of nucleic acids in the entire genome of an organism. The genome comprises all coding and non-coding nuclear DNA in a cell and also includes the uniparentally inherited organelle DNA, i.e., in the plastids and mitochondria. Different sequencing technologies and platforms are available (see e.g. overviews in Holliday et al. 2019; Porter and Hajibabaei 2018). Especially Illumina sequencing platforms (Illumina, San Diego, CA) are widely used. Typically, the DNA of an individual is extracted and fragmented. Fragmentation can be achieved e.g. by acoustic shearing using an ultrasonicator. The fragments are size selected prior to adapter ligation. The adapters serve both as a connection to the Illumina sequencing flow cell and as primers for amplification prior to sequencing and during the sequencing reaction itself. The sequence reads of the DNA fragments are then bioinformatically processed. First, the reads are quality filtered and can subsequentially be aligned to a reference genome or are used for de novo genome assembly. For de novo genome assembly, ideally, sequences with different lengths (long reads from more error-prone technologies and short reads) are combined to maximize coverage and avoid gaps. In the last years, with decreasing sequencing costs, more and more tree species reference genomes are becoming available and are gathered in the TreeGenes database (https://treegenesdb.org/; Falk et al. 2018) where currently 29 genome and draft genome assemblies of tree species are stored, mostly of broadleaf trees of economic interests (by uses of wood, resins, or fruits, or as ornamentals), but also 6 gymnosperms including the evolutionary old genera *Gnetum*, *Gingko* and *Sequioa*. Also, other non-tree-specific databases like Phytozome (https://phytozome-next.jgi.doe.gov/; Goodstein et al. 2012) contain valuable genome information of tree species.

Comparative studies between herbaceous plants and tree genomes identified whole genome duplication events, major chromosome rearrangements, and expansions in gene families especially involved in the expression of tree-specific traits, such as wood formation (reviewed in Plomion et al. 2016). Rates of molecular evolution are typically lower in long-lived and outcrossing tree species compared to herbaceous species (Smith and Donoghue 2008). While monocot genomes (average genome size of 9.4 Gbp [std. dev. 12.3]; Pellicer and Leitch 2020) and eudicot genomes (average genome size of 2.4 Gbp [std. dev. 3.9]; Pellicer and Leitch 2020) are usually small or medium-sized, gymnosperm genomes, particularly those of conifers (e.g., *Pinaceae* average genome size of 23.4 Gbp [std. dev. 5.6]; Pellicer and Leitch 2020), are much bigger which was most likely caused by long insertions of repeated elements, such as repeat-retrotransposons and a lack or slower rate of efficient DNA repair mechanisms (Cossu et al. 2017; Morse et al. 2009; Nystedt et al. 2013; Wegrzyn et al. 2013). Predictions of gene numbers in sequenced tree species are around 30,000 to 40,000 regardless of the genome size (Cao et al. 2022).

Once a reference genome is available, higher numbers of samples can be re-sequenced and aligned to this reference to determine sequence variation between individuals, such as SNPs, insertions, and deletions. In the same sequencing run, several samples can be pooled and, if necessary, later bioinformatically separated based on individual index sequences which can be added to the adapters during library preparation (Holliday et al. 2019). Especially for natural populations with low linkage disequilibrium or species with a small genome size for which no genome-wide genotyping assays have been developed, whole genome re-sequencing can now produce valuable data sets.

## Genome complexity reduction

Due to the large genome size of some tree species, the whole genome sequencing methods described in the previous section are still too expensive for many projects. Often, whole genome sequencing or re-sequencing is also not the best approach for the given research question. For instance, a researcher may only be interested in the coding part of a genome, and hence, sequencing the complete genome including non-genic and non-coding parts may not be efficient. Therefore, different techniques for genome complexity reduction, such as sequence capture or genotyping-by-sequencing (GBS), have been developed.

Sequence capture allows the targeting and enrichment of specific regions within a genome. This approach is based on hybridization between the DNA regions of interest and specifically designed oligonucleotide baits (Gasc et al. 2016; Holliday et al. 2019). The obtained libraries are afterwards sequenced using next-generation sequencing platforms. Thereby, different DNA regions can be targeted. For instance, researchers may be interested in re-sequencing specific DNA regions or they specifically target exonic regions—a technique known as exome capture. Exome capture has been used in recent years to detect and analyze genetic variation in coding regions of tree genomes including large and complex conifer genomes. Exome capture baits can be designed based on genome or transcriptome data. For instance, Lu et al. (2016) used exome capture to identify SNPs in 375 loblolly pine trees (*Pinus taeda*) of a mapping population in the USA and to investigate population structure and linkage disequilibrium. The authors identified more than 972,000 SNPs and detected two distinct subpopulations referring to different geographic origins of the trees. In cases, where no reference genome is available for the target species, also genomic resources of closely related species can be used for bait design. For instance, Capblancq et al. (2020a) successfully used reference transcriptomes of *Picea glauca* to design exome capture baits for the related tree species *Picea rubens*.

However, despite the advantages of sequence capture for genome complexity reduction, such as the specific selection of target regions and the generation of data sets with less missing data compared to other methods, it is also more expensive and sequence information is necessary for probe design (Holliday et al. 2019). Therefore, restriction enzyme-based methods, such as genotyping-by-sequencing (GBS) (Elshire et al. 2011) or restriction site-associated DNA sequencing (RAD-seq) (Baird et al. 2008), are more often applied in forest genetics research. GBS and RAD-seq have been used as umbrella terms describing different methods that use restriction enzymes to guide genome complexity reduction and sequencing (Parchman et al. 2018). These methods might differ regarding the amount of reliably identified SNPs (Ulaszewski et al. 2021). Since no reference genome is necessary for these methods and they are comparably inexpensive, they can be used for SNP identification in non-model tree species with large sample sizes. GBS and RAD-seq methods provide valuable data for a variety of applications, such as phylogenetics (Hipp et al. 2020), genetic mapping (Konar et al. 2017), or different population/landscape genetics research questions (Johnson et al. 2017; Martins et al. 2018; Sun et al. 2016).

A modification of the RAD-seq method, which combines bisulfite treatment of DNA with RAD-seq, makes it possible to analyze genome-wide epigenetic patterns (DNA methylation) in forest tree species (e.g., bsRAD-seq, Trucchi et al. 2016). The bisulfite treatment converts non-methylated cytosine into uracil (Frommer et al. 1992; Henderson et al. 2010). By comparison with untreated DNA sequences, DNA methylation patterns can be identified (bisulfite sequencing can also be applied in whole genome sequencing studies). Gugger et al. (2016) used reduced-representation bisulfite sequencing for the identification of epigenetic variants in *Quercus lobata* and association analysis of these variants with environmental variables. Recently, an alternative approach (enzymatic methyl-seq (EM-seq)) has been developed to investigate the methylome without bisulfite sequencing leading to a lower GC bias and longer sequencing reads (Williams et al. 2019). In this method, the conversion of non-methylated cytosine to uracil is conducted by enzymatic treatment of the DNA.

## RNA-Seq

RNA sequencing (RNA-seq) is used to determine genome-wide gene expression. It provides a direct measurement of RNA transcript abundance and allows a simultaneous identification and quantification of sequences (Weber 2015). Since this method does not need prior sequence information, it can be used for the analysis of transcriptomes (i.e., complete sets of transcripts in a cell (Wang et al. 2009)) of tree species without or with only restricted genomic resources available. In cases where no reference transcriptome is available, usually a de novo transcriptome assembly is conducted (see below). After RNA extraction, reverse transcription is used to convert RNA into cDNA, which is used for library preparation and sequencing.

RNA-seq can be used to get insights into gene expression in general and in response to, for example, different biotic and abiotic stressors to investigate the genetic basis of specific traits. For instance, Harper et al. (2016) used RNA-seq to identify SNPs and associate them with damage scores of European ash (*Fraxinus excelsior*) trees which were affected by the ash dieback disease caused by the invasive fungal ascomycete pathogen *Hymenoscyphus fraxineus*. The authors found markers associated with damage levels and identified SNPs which were successfully used to identify trees with a low level of susceptibility to the disease. Other studies used RNA-seq to investigate gene expression in response to stressors, such as drought, frost, high salt concentration, or herbivory, in several tree species (e.g., Breidenbach et al. 2020; Chaires et al. 2017; Fox et al. 2017; Kersten et al. 2013; Müller et al. 2017; Wu et al. 2019). Thereby, trees (mostly seedlings/saplings) under controlled conditions (e.g. climate chambers) are often experimentally divided into two groups, of which one is treated and the other one is untreated (control samples). Based on RNA-seq data obtained in these groups, genes are identified, which are significantly differentially expressed between treatment groups, and hence, potentially involved in stress response. Another attractive approach is the identification of gene expression networks that are conserved among species. This was done by Zinkgraf et al. (2020) for the trait “wood formation”. The authors used RNA-seq to investigate gene expression related to wood formation in 13 different tree species and identified orthologous genes whose co-expression relationships were maintained across species (Zinkgraf et al. 2020).

Further, transcriptome data are valuable sources for the development of genetic markers, such as microsatellites or SNPs. For species with scarce genomic information, RNA-seq can provide valuable resources, such as the identification of candidate genes for specific traits or reference transcriptomes. For the establishment of reference transcriptomes, preferably several different tree tissues should be sequenced, since tissues can differ regarding gene expression. For instance, Guerrero-Sanchez et al. (2017) established a reference transcriptome for holm oak (*Quercus ilex*) based on a sample consisting of homogenized tissue from acorn embryos, leaves, and roots. Transcript abundance is dramatically varying within a transcriptome. This means that more abundant transcripts are recurrently sequenced, while rare transcripts may not reach the necessary sequencing depth for analysis. Therefore, normalization of cDNA equalizing transcript abundance can be meaningful prior to sequencing, for samples that will be used for de novo transcriptome assembly, albeit it can be associated with drawbacks, such as high costs or erroneous removal of genes (Honaas et al. 2016). Normalization of cDNA, for instance, has been applied in transcriptome studies of European beech (*Fagus sylvatica*) and pedunculated oak (*Quercus robur*) and led to low redundancy transcriptome assemblies (Müller et al. 2017; Tarkka et al. 2013).

RNA-seq has also been used to gain insights into epigenetic mechanisms in tree species by analyzing small RNAs (e.g., Liu and El-Kassaby 2017; Yakovlev et al. 2016; Yakovlev and Fossdal 2017), which are involved in the control of gene expression and potentially in several epigenetic mechanisms (Sow et al. 2018). For instance, Yakovlev and Fossdal (2017) used RNA-seq to screen embryogenic tissues of Norway spruce that was produced under different temperatures for small RNAs. The authors found 654 microRNAs (miRNAs) that were differentially expressed with respect to temperature levels. They concluded that fine-tuning of miRNA production might be involved in developmental regulation and epigenetic memory formation in Norway spruce (Yakovlev and Fossdal 2017).

## Case studies—application of genetic methods in tree conservation genetics, tree breeding, and association analysis

### Conservation genetics

In conservation genetics, researchers aim at understanding the evolutionary dynamics affecting the genetic variation, particularly in rare populations and species to preserve them from extinction by providing guidance for conservation or restoration management. Genetic markers, such as isozymes or organelle and nuclear microsatellites, have traditionally been employed to determine the levels of genetic diversity, reveal phylogeographic patterns, study the demographic history, and assess the effect of land use changes. Genetic markers were used to define populations of conservation priority (“evolutionary significant units” (Moritz 1994) or “management units” (Palsbøll et al. 2007)) mostly in threatened or rare species. Most European forest tree species show high levels of genetic variation (but see e.g. *Pinus pinea* (Mutke et al. 2010)) and are not considered threatened or rare. Nevertheless, the conservation of forest genetic resources has a long tradition and has led to the creation of the European Forest Genetic Resource Program (EUFORGEN; http://www.euforgen.org/). Nowadays, global change is rapidly changing the environmental conditions and thereby is raising concern to develop improved sustainable conservation approaches to maintain high levels of genetic variation and the dynamics of evolutionary processes to promote range-wide adaptation, and especially in peripheral populations (Fady et al. 2016). Combining knowledge obtained from neutral genetic markers and from relevant quantitative traits in shared experimental plantings (so called "common gardens") can improve the ability of defining evolutionary units for conservation, an approach e.g. employed in maritime pine to improve the dynamic conservation program (Rodríguez-Quilón et al. 2016). However, common gardens spanning the complete distribution range of a species are still lacking for most forest tree species and often peripheral populations are underrepresented and the common gardens are seldomly sufficiently replicated (Fady et al. 2015). Genome-wide genetic markers and new computational tools are therefore promising to identify loci showing functionally important differentiation also in non-model species and use this information for improved conservation recommendations. Fitzpatrick and Keller (2015) were able to incorporate genomic markers in a niche modelling framework and uncovered gene-environment relationships in balsam poplar (*Populus balsamifera*) providing relevant information for the vulnerability of populations under a climate change scenario. Recently, several genomic off-set studies raised concern by revealing populations mal-adapted to the predicted future climatic conditions (reviewed in Capblancq et al. 2020b). Conservation genomic initiatives combining whole genome re-sequencing of multiple species and landscape genomic analyses can reveal regions with high intraspecific diversity and corridors connecting these regions, as well as areas of conservation concern (Shaffer et al. 2022).

### Breeding

Several properties of forest tree species, such as long generation times, late flowering, or weak juvenile-mature correlations, make forest tree breeding difficult (Grattapaglia et al. 2018). Conventional breeding is a slow process, in which several years are necessary for breeding (5–15 years) and progeny testing (3–15 years) (Isik 2014). Therefore, genetic methods are needed to shorten breeding cycles. One such proposed method is marker-assisted selection (MAS), in which genetic markers with large effects are identified that are associated with the trait of interest (see QTL mapping below) and could be used for screening seedlings for planting/breeding. Nevertheless, MAS was not very successful in forest tree breeding, mainly because identified QTL only explained a small amount of phenotypic variation (Isik 2014). Another method called genomic selection (GS) has become possible due to the development of high-throughput sequencing techniques and is very promising. Instead of identifying discrete marker-trait associations, in GS large numbers of SNPs that cover the whole genome are jointly analyzed to predict breeding values (Grattapaglia et al. 2018; Isik 2014; Meuwissen et al. 2001). Often a few thousand SNPs are used in GS studies with forest tree species, albeit numbers are varying between ca. 2,500 to 69,000 (Chen et al. 2018b). For the identification of SNPs, often methods for the reduction of genome complexity, such as exome capture or GBS (see section “Genome complexity reduction”), are employed. For instance, Chen et al. (2018b) used exome capture to identify SNPs in Norway spruce for GS. In total, 116,765 SNPs were used for evaluating the influence of different parameters, such as the relatedness of the trees, size of the training and validation set, or the number of SNPs, on the accuracy and predictive ability of GS for growth and wood quality traits. The study indicates that GS would reduce the time needed for a breeding cycle in breeding programs that rely on long-term progeny testing. Further, Chen et al. (2018b) concluded that ca. 8,000 SNPs would be required for GS in a full-sib (sibling) family of sufficient within family size (16 trees for growth and 12 trees for wood quality traits).

The described methods can also be used in breeding programs aiming at increasing the resistance of trees against pests and pathogens (Naidoo et al. 2019). For instance, Nvsvrot et al. (2020) used QTL mapping in combination with genome re-sequencing to identify variation within an *R* (resistance) gene which is associated with variation in leaf rust disease resistance in poplar. However, resistances sometimes break down as exemplified in 1994 in France by the poplar Rmlp7 resistance against the obligate biotrophic *Melampsora larici-populina* (Persoons et al. 2017). Breeding for loss of function *S* (susceptibility) genes may be even more promising, since it may lead to more durable and broad-spectrum resistance (Pavan et al. 2010; van Schie and Takken 2014). Analyses of *S* genes have been conducted for resistance to powdery mildew fungi in tree species, such as rubber trees (*Hevea brasiliensis*) or black cottonwood (*P. trichocarpa*), or related to the plant-damaging fungal-like oomycete *Phytophthora* spp. and to *Cryphonectria parasitica* in sweet chestnut (*Castanea sativa*) (Filiz and Vatansever 2018; Liyanage et al. 2020; Pavese et al. 2021). One of the most prominent examples of increasing pest resistance in forest tree species is the breeding of American chestnut (*Castanea dentata*) trees for tolerance against chestnut blight, a disease caused by the bark fungus *C. parasitica* which was accidentally introduced to the USA around 1900 (Jacobs et al. 2013; Merkle et al. 2007) and led to the near extinction of American chestnut (Aucott and Parker 2021; Lovat and Donnelly 2019). Conventional backcross breeding was used to incorporate blight resistance from Chinese chestnut (*Castanea mollissima*) into American chestnut. This resulted in backcrossed hybrids that have a greater blight resistance than pure American chestnuts, but less than the resistance displayed by F_1_ hybrids of Chinese and American chestnuts (Aucott and Parker 2021). Higher resistance of American chestnut was obtained using genetic engineering (Newhouse et al. 2014; Zhang et al. 2013). Specifically, an oxalate oxidase gene (*OxO*) from wheat was transferred into American chestnut via *Agrobacterium*-mediated transformation (Carlson et al. 2022; Onwumelu et al. 2022; Polin et al. 2006). Oxalic acid is produced by the fungus *C. parasitica* and destroys chestnut tree bark tissues, among others by decreasing the intracellular pH and the lignin content (Lovat and Donnelly 2019; Welch et al. 2007). OxO catalyzes the degradation of oxalate in H_2_O_2_ and CO_2_ and speeds up the oxidation of oxalic acid in chestnut which protects lignin from degradation (Aucott and Parker 2021; Chang et al. 2018; Welch et al. 2007). Since OxO does not kill the fungus but only mitigates its impact on plant tissue and lignin loss, the tolerance of genetically modified American chestnuts might be quite sustainable (Chang et al. 2018). In 2020, a deregulation petition (Newhouse et al. 2020) for genetically engineered blight-tolerant American chestnut trees (“Darling 58”) was submitted to the United States Department of Agriculture (USDA).

In general, genetic engineering can be used to alter traits of interest in tree species, such as growth, wood properties, abiotic and biotic stress tolerance, or reproduction control (see Chang et al. (2018) for an excellent review). Nelson (2022) argues in his recent review that genetic engineering is not a shortcut to tree improvement and conventional tree breeding and genetic engineering should be seen as complementary methods. Despite the use of genetic engineering in research, the commercial use of genetically modified trees is very limited (Chang et al. 2018). China was the first country that released genetically modified forest trees for commercial use. In 2002, insect-resistant *Populus nigra* trees that contained a modified *Cry1Ac* toxin gene from *Bacillus thuringiensis* (*Bt*) were used for commercial plantations in this country (Chang et al. 2018; Hu et al. 2014; Zheng 2010). In 2015, the company FuturaGene obtained permission to release a transgenic *Eucalyptus* with enhanced wood production for commercial use in Brazil (Anonymous 2015).

### Association genetics and outlier detection

Association genetics aims at identifying genotypes significantly associated with phenotypic traits or environmental variables. Genome-wide association studies (GWAS) using dense genomic data are of course best suited to detect loci under selection (see below), but in many non-model species, genomic resources are still limited.

One of the earliest approaches in association genetics is Quantitative Trait Locus (QTL) mapping. QTL mapping requires the availability of large segregating full-sib families. In crop plants and herbaceous plants with short generation cycles, F_2_ backcross families and recombinant inbred lines have been used for the construction of genetic linkage maps and QTL mapping (Tanksley 1993; Xu et al. 2017). In contrast, outcrossing forest trees with very long generation times are characterized by a high level of individual heterozygosity enabling the use of F_1_ full-sib progenies to construct separate male and female and joint linkage maps using a two-way pseudo-testcross strategy (Grattapaglia and Sederoff 1994). In model tree species with high economic importance, also intra- and interspecific backcross families have been generated and used for QTL mapping (Bdeir et al. 2017; Muchero et al. 2013). QTLs have been identified for a wide range of growth-related and potentially adaptive traits, such as drought tolerance, phenology, wood quality, and disease resistance (Brendel et al. 2008; Drost et al. 2015; Kubisiak et al. 2013; Scotti-Saintagne et al. 2004). Genome-wide sequencing methods including whole genome resequencing are gaining importance in QTL mapping in trees (Marinoni et al. 2020). While the generation of genomic data is no longer a limiting factor for some model tree species, additional efforts are needed in high precision and high throughput phenotyping to dissect the genetic basis of complex phenotypic traits (Sideli et al. 2020; Virlet et al. 2015). Also, allelic variation in the crossing parents and the comparatively low resolution of the QTL mapping approach limits the detection of genes underlying trait-specific QTLs, high precision mapping resulting in QTL regions that still contain several hundred genes (Bdeir et al. 2017). Complex traits, such as the timing of bud burst are generally controlled by many genes with individually small effects on the phenotype and major QTLs explaining more than 20% of the phenotypic variation are comparatively rare (Brendel et al. 2008; Marinoni et al. 2020).

Another often applied association genetics approach is the candidate gene approach that targets loci in coding regions of the genome with possible relevance for the phenotypes under study. Those studies revealed loci significantly associated with e.g. wood properties and height growth (e.g., Cabezas et al. 2015; González-Martínez et al. 2007) or ecologically important traits, such as cold hardiness (e.g., Eckert et al. 2009; Holliday et al. 2010) or drought resistance (Cuervo-Alarcon et al. 2021). To avoid the confounding effects of environmental variation typically found in natural populations, most of these studies were conducted in field trials or under experimental conditions. However, in some cases, for example, when selection pressure is strong, promising candidate genes are known and highly heritable plant traits are targeted, association studies can also be conducted successfully in natural populations (Budde et al. 2014; Caré et al. 2020). Environmental association studies also revealed correlations between adaptive genotypes/phenotypes or allele frequencies for particular loci and environmental clines, such as temperature or drought gradients (Bergmann 1978; Eckert et al. 2010; Jaramillo-Correa et al. 2015). Another approach is the detection of outlier loci showing stronger divergence than expected under a neutral model when comparing e.g. individuals growing in contrasting habitats (Beaumont and Nichols 1996). An interesting example is the strong divergence at several candidate genes in *Eperua falcata*, a tropical forest tree growing on a mosaic of seasonally flooded bottomlands and seasonally dry terra firme soils in close vicinity (Audigeos et al. 2013).

Genome-wide association studies (GWAS) aim to identify associations between a panel of genome-wide genetic markers and phenotypes (reviewed in Korte and Farlow (2013)). Usually, a large number of individuals is used. One big advantage of GWAS is that no pedigree information is needed. Because in the first step genetic markers need to be identified, which are subsequently associated with the trait of interest, often several genetic methods discussed in this review are combined. For instance, Ćalić et al. (2017) used RNA-seq to identify SNPs related to the fatal beech bark disease, a pathosystem caused variously by interplays of different bark scales (*Xylococculus betulae*, *Cryptococcus fagisuga*), pathogenic *Neonectria* fungi and abiotic factors (Cale et al. 2017). The SNPs were subsequently used to genotype 514 trees from North America that were susceptible or resistant to the beech bark disease. Afterwards, a GWAS was conducted, in which SNPs were associated with disease scores. The authors also conducted linkage mapping in a full-sib family. Finally, four highly significant SNPs from a single gene were identified that were located on chromosome 5. Thus, a single locus with a major effect was identified that contributed to resistance against beech bark disease (Ćalić et al. 2017). Also, genome-wide genetic markers obtained by GBS can be employed in GWAS studies. Parchman et al. (2012) were one of the first to apply this method in natural populations of lodgepole pine and could identify loci significantly associated with fire adaptive traits. Using only 11 loci, they could explain 50% of the phenotypic variation in serotiny. Pooling individuals in groups for whole genome sequencing can lower the sequencing costs. This approach was adopted by Stocks et al. (2019) to identify loci significantly associated with the susceptibility of common ash trees to ash dieback caused by the invasive pathogen *H. fraxineus*. They pooled all healthy and all diseased common ash trees from each population origin in separate sequencing pools, by that obtaining allele frequencies for genome-wide genetic markers per population and susceptibility group. Subsequently, a GWAS identified 3149 SNPs significantly associated with ash dieback susceptibility, revealing a highly polygenic trait.

New genome-editing techniques, especially the CRISPR/Cas9 system, are promising methods to investigate and alter gene functions in tree species (Cao et al. 2022). CRISPR/Cas9 has been used to knockout (KO) target genes in poplar and led to a better understanding of traits such as bud outgrowth, secondary cell wall formation, or sex determination (Bruegmann et al. 2019; Muhr et al. 2018; Müller et al. 2020b; Takata et al. 2019). In the future, this method may become a valuable tool to confirm candidate gene functions revealed by population genetic methods such as GWAS (Fernandez i Marti and Dodd 2018).

## Fungal communities associated with trees

### Fungal guilds living in interactions with trees

Saprotrophs, symbionts, pathogens, and endophytes (Fig. 1; Box 2) are on a functional scale and based on resource use differentiated into distinct main ecological guilds, here i.e. groups of fungal species that exploit the same resources or different resources in a related manner (Adnan et al. 2022; Langer et al. 2021; Nguyen et al. 2016b; Talbot et al. 2015; Weißbecker et al. 2018; Zanne et al. 2020). Newly emerging as other guilds of ecological relevance are distinct spatially distributed communities of fungal epiphytes sitting on aerial surfaces of plant organs, e.g. on the phylloplane of leaves (Bahram et al. 2022; Ding et al. 2022a; Fonseca et al. 2022; Gomes et al. 2018; Howe et al. 2016; Liber et al. 2022; Sun et al. 2021; Vacher et al. 2016), on surfaces of flowers (anthosphere) and fruits (carposphere; Bill et al. 2022) or on the bark of stems (caulosphere; Cook et al. 2022), with potential functions such as of host defense on the tree or advanced inoculation of plant litter for decay directly when fallen (Box 2). Without these various possible microbial confrontations, trees will suffer in different ways.

**Box 2** Fungal lifestyles in ecological relation to trees and forests

**Table:** 

Lifestyle	Description
Saprotroph	A heterotrophic organism that feeds on the extracellular (enzymatic) decomposition of dead organic matter to obtain energy, carbon, and nutrients for growth and development by absorbing the nutrients released from the decaying material Of particular ecological significance in forest ecosystems are plant-litter-degrading fungi and wood decay fungi that act in nutrient cycling
Symbiont	An organism that lives in a mutual relationship with another living organism for the benefit of both Of imminent significance for trees are mycorrhizal fungi that in association with roots help the plant host in nutrition, may protect against abiotic and biotic stresses, and may also influence positively the host soil biology and chemistry Other fungal symbionts emerge from the endophyte concept living biotrophic in distinct alive plant organs, for instance in tree leaves, with novel supportive roles for the host, e.g. help to withstand biotic and abiotic stresses
Pathogen	Any organism that causes a type of disease to a host Biotroph living without killing the infested host tissue is distinguished from hemi-biotroph with an initial biotrophic phase followed by the killing of infested host tissue for consumption and from necrotroph where the pathogen kills and lives from the killed tissues of the host Any organ of a tree may be negatively affected by a wealth of possible fungal diseases
Endophyte	An organism that lives neutrally inter- or intracellularly (endobiotic) within a plant and is nourished by its host without causing any overt disease or reduction in the hos'´s fitness Any organ of a tree may be populated by fungal endophytes From an ecological perspective, an endophyte is however unlikely to be entirely neutral, but may live symbiotically in unrecognized mutualistic interactions with its host, or perhaps as a commensal gaining food and water as uncompensated service from the host, or be subtle pathogenic by weakening the host through taking energy and nutrients while environmental stress signals may render the lifestyle to obvious pathogenic with clear symptoms of disease
Epiphyte	An organism that grows on the surface of a plant and derives its moisture and nutrients from the air Fungi transported e.g. by insect vectors or from the aeromycota to phyllospheres shape host-, organ- and vertical-stratification-specific but yet little-analyzed communities on the aerial surfaces of tree organs

Saprotrophs of recalcitrant dead organic material (mostly kinds of above and belowground plant litter and deadwood, each with its own shaped sets of fungal communities; Fig. 1) will ensure humus generation and, of imminent importance for the trees, the recycling of bound organic carbon and further elements needed for new plant growth (Adnan et al. 2022; Bödeker et al. 2016; Floudas et al. 2012, 2020; Kües et al. in prep.). Dead plant litter is complex and diverse in composition, context-dependent by habitat and plant source. In forests, plant litter comes in large parts from leaves, needles, twigs, bark, wood chips, fine roots, fruits, or also seeds as dying or dead debris of trees with different degrees of lignification, make-up of antimicrobial compounds and water activity (a_w_) values (Argiroff et al. 2023; Chomel et al. 2016; Freschet et al. 2013; Lonsdale 1988; Ochoa-Hueso et al. 2019). Aboveground litter decomposition and progressive humus generation on the soil surface (Horizon O; Fig. 1) and belowground more stable soil organic matter (SOM) formation with C and N in mineral soil (Horizon A, Horizon B; Fig. 1) involve complex interplays of changing microbial communities (fungi, bacteria, and others; Bai et al. 2021; Kües et al. in prep.) and detritivorous soil meso- and macrofauna with multiple pathways of turnover and accumulations of intermediate microbial and faunal transformation products, which is further influenced in interactions with the vegetation, with soil properties and (bio)chemistry, and also by climate parameters (Prescott and Vesterdal 2021; Veldkamp et al. 2020). What is more, coarse wooden material degradation will prepare fermented porous-structured moisture-retaining nutritious grounds for better germination and rooting of seedlings and anchorage of developing trees (Fukasawa et al. 2017, 2020; Stroheker et al. 2018) and provides also habitat for saproxylic communities (Fukasawa 2021). Seed beds of decaying logs or stumps may harbor less fungal seed pathogens and they can provide favoring associations of N_2_-fixing bacteria with specific selections of mycorrhiza-promoting fungi (Huusko et al. 2015; Izumi et al. 2006; Tedersoo et al. 2008; Willis and Walters 2018). Saprotrophs are mostly generalists with regard to substrates, although fungal species heterogeneity exists, e.g. in the decay of softwood and hardwood and in wood decomposition efficiencies due to specialization of fungal communities, which are characteristically adapted through the plant community above them (Awad et al. 2019; Chaithaisong et al. 2022; Kües et al. in prep.; Prescott and Grayston 2013; Purahong et al. 2018, 2019; Tedersoo et al. 2014; Yang et al. 2021).

On the contrary, mycorrhizal fungi and pathogenic fungi are nutritionally dependent, to varying degrees, on the specific narrower or broader ranges of their living sessile tree hosts and their photosynthetic products (van der Linde et al. 2018; Weißbecker et al. 2018). Tree species in boreal, temperate, and Mediterranean zones with colder and dryer climates and trees at higher latitudes undergo mutualistic interactions most often with ectomycorrhizal (ECM) species, either ascomycetes or most often basidiomycetes. In warm humid aseasonal climates, arbuscular endomycorrhiza of *Glomeromycota* dominates on trees (Adnan et al. 2022; Soudzilovskaia et al. 2019; Steidinger et al. 2019). Mycorrhizal fungi will exist both in soil and in planta, with possible residual capabilities of saprotrophic decomposition of plant litter. They help to provide the trees with water and nutrients, especially N and P, through mycorrhizae formed with host roots and receive mutual nourishment (symbiotrophy) organic carbon resulting from photosynthates (Fig. 1). Forest soils show a high degree of stratification (Fig. 1), with C:N ratios decreasing with increasing litter age and soil depth (Bai et al. 2021; Lindahl et al. 2007) and changing fungal guilds of saprotrophic (SAP) fungi found typically more in the upper organic layers and, more or less spatially separated, ECM species underneath in the mineral soil layers (Khokon et al. 2021; Lindahl et al. 2007; Fig. 1). The different decomposing strategies of the fungi in the organic layer have further consequences, particularly for N retention in and stabilization of SOM (Baskaran et al. 2019; Boberg et al. 2011, 2014; Hasby et al. 2021; Mrnka et al. 2009, 2020). It is noteworthy that the mycorrhizal fine roots are relatively short-lived. Combinations of fungal ECM and saprotrophic fungi in the rhizosphere control the speed of decay of senescent absorptive fine roots as a primary source of SOM (Angst et al. 2021; Argiroff et al. 2023; Jackson et al. 2017; Kües et al. in prep.). Mycorrhizae mediate resistance and defense reactions against belowground and aboveground biotic and abiotic threats and confer protection against soil pollution. By hyphal growth and mycelial networking in the soil, the fungal symbionts moreover promote particle aggregation in soil (Adnan et al. 2022; Dreischhoff et al. 2020; Genre et al. 2020; Leyval et al. 1997; Schützendübel and Polle 2002; Sivaprakasam Padmanaban et al. 2022; van der Heijden et al. 2015). Habitually negatively perceived due to the damages and losses they cause to individual trees, plantations, or forests, pathogens might live biotrophic on alive host cells or necrotrophic on host tissues killed by them (Fig. 1). However, microbial pathogens can positively assist ecosystem functioning in turnover of plant communities for renewal and keeping species balances, and they are key drivers of ecosystem’s biodiversity, much like animal tree consumers (Adnan et al. 2022; Gilbert 2002; Hawkins and Henkel 2011; Kües et al. in prep.; Schuldt et al. 2017; Zeilinger et al. 2015).

Fungal root (mycorrhizal or endophytic), leaf, and other symbionts can also drive plant community biodiversity, e.g., by strengthening their respective hosts (Rodriguez et al. 2009; Zanne et al. 2020). Endophytes grow symptomless in plant tissues (Fig. 1), most often intercellularly or possibly also intracellularly, and maybe organ-specific or, as recently evidenced, also may grow throughout a tree (Küngas et al. 2020; Rodriguez et al. 2009). As part of multifunctional changing lifestyles, many exist also widely distributed in soils as saprotrophs. Endophytes have emerging mutualistic roles in planta in host protection against pests and pathogens, e.g., by antagonistic bioactive secondary metabolite production, by mycoparasitism of adverse pathogens, and by occupying an in planta niche that becomes then unavailable for invasion of pathogens. Fungal endophytes may confer abiotic stress tolerance and some promote plant growth by plant hormone production (Adnan et al. 2022; Barge et al. 2022; Eberl et al. 2019; Jia et al. 2020; Rabiey et al. 2019; Rodriguez et al. 2009; Zanne et al. 2020). Others are latent and turn as opportunists into a pathogenic lifestyle once host tissues become stressed or when they senesce (Hardoim et al. 2015; Rabiey et al. 2019) or they are latent decomposers awaiting the host death as a resource for plant material consumption (Parfitt et al. 2010). In a broader interpretation of a lifestyle that has recently been termed viaphytism (Nelson et al. 2020), certain decomposing fungi can use as endophytes organs of the plant host as a refuge to overcome periods of own environmental stress. For example, such species may exist as temporary foliar endophytes to then enhance their own spread by leaf and needle fall to the forest floor providing more versatile woody substrates to them for saprotrophic hyphal growth (Nelson et al. 2020; Vaz et al. 2020). Functions of still other endophytes remain elusive (Gehring et al. 2020; Rodriguez et al. 2009), or may they simply be commensals nurtured by a host without recognizable impacts on their feeder (Langer et al. 2021; Terhonen et al. 2019; Zanne et al. 2020)? In stricter definition, a "true endophyte" is thus a "commensal that does not decrease the fitness of its host and cannot switch to a different lifestyle". Some of them develop an obligatory interaction with the host, for instance, the sapwood ascomycete *Xylona heveae* with the rubber tree *H. brasiliensis*. Such strict endophyte can then depend under circumstances on horizontal transmission through flying insects as vectors (horizontally transmitted endophyte, HTE; Gazis et al. 2016). Leaves of woody plants can be especially heterogenous in assembling communities of many different HTEs, often with broad host-ranges. Foliar HTEs of woody plants are typically horizontally transmitted by wind, rain, or also vectoring animals, maybe from senescent tissues or dead fallen leaves after hyphal fragmentation or any type of aerial spore production (aeromycota; Fig. 1; Rodriguez et al. 2009). Tree host genotypes and specific leaf traits (content of cell wall polysaccharides, flavonoids, terpenoids, also leaf nutrients, and leaf mass per area) act as non-random filters on HTE colonization by fungal taxa (González-Teuber et al. 2020; Redondo et al. 2022). According to a GWAS study in spruce, genotypic QTL variation influenced dormant vegetative bud fungal endophytic phyllosphere and latent pathogen communities (Elfstrand et al. 2020).

### Fungal genomes and transcriptomes

Genomic techniques as explained above apply to trees as to their associated fungi, as isolated organisms or in interactions (Nilsson et al. 2019; Stewart et al. 2018). With their much smaller genomes (usually somewhere between 20 and 100 Mb; Mohanta and Bae 2015), the fungal genomes are however easier to address, with faster-accumulating information. In the last decade, many fungal genomes were fully established in large-scale sequencing projects for deeper functional insight (many to be found on the MycoCosm portal of the Joint Genome Institute, JGI in Walnut Creek, California; https://mycocosm.jgi.doe.gov/mycocosm/home; Grigoriev et al. 2014). Larger portions of fungal genes were initially expert-annotated, but later, under the support of also sequenced transcriptomes and with machine learning, the about 10,000 to > 20,000 protein-encoding genes per species became more and more reliably automatically annotated by gene prediction programs. With regards to trees, sequenced genomes were mostly of basidiomycetous wood decay fungi (e.g., Fernandez-Fueyo et al. 2012; Floudas et al. 2012; Hori et al. 2014; Levasseur et al. 2014; Martinez et al. 2004, 2009), including some root and stem rotting pathogens (Akulova et al. 2020; Kües et al. 2015; Ohm et al. 2010; Olson et al. 2012). Genomes of some other types of asco- and basidiomycetous pathogens, such as some on leaves (Dhillon et al. 2015; Duplessis et al. 2011a; Zhu et al. 2012) and of wilt-, blight- and cancer-pathogens in bark and xylem are available, including those of several insect-associated fungal species (Alamouti et al. 2011; Comeau et al. 2015; Crouch et al. 2020; Demené et al. 2022; Dhillon et al. 2015; Ibarra Caballero et al. 2019; Sbaraini et al. 2017; Schuelke et al. 2017; Stauber et al. 2020; Stenlid et al. 2017; Yin et al. 2015). There is also a growing collection of genomes of symbiotic ECM species (e.g., Kohler et al. 2015; Lofgren et al. 2021; Looney et al. 2022; Martin et al. 2008, 2010; Martino et al. 2018; Miyauchi et al. 2020b; Peter et al. 2016; Wagner et al. 2015), some ericoid mycorrhizal ascomycetous fungi (Kohler et al. 2015; Martino et al. 2018; Perotto et al. 2018), an arbuscular mycorrhizal fungus from the conifer *Cryptomeria japonica* (Matsuda et al. 2021), and of a few tree endophytes (Gazis et al. 2016; Knapp et al. 2018; Schlegel et al. 2016) while plant litter decay fungi and in general saprotrophic soil fungi have so far rather been neglected (Barbi et al. 2020).

RNA-seq of isolated transcriptomes and determination of proteomes support the identification of genetic functions of these fungi crucial for their interactions with host trees (see e.g., Chaudhary et al. 2020; Daguerre et al. 2017; Doré et al. 2017; Duplessis et al. 2011b; Lorrain et al. 2018; Marqués-Gálvez et al. 2021; Peter et al. 2016; Plett et al. 2014; Tisserant et al. 2011) and in complex organic substrate degradation (e.g., Alfaro et al. 2020; Arntzen et al. 2020; Barbi et al. 2020; Janusz et al. 2018; Kuuskeri et al. 2016; Miyauchi et al. 2020a; Zhang et al. 2019). In some instances, it is almost only RNA-seq that could reveal probable relevant differences by specific gene expression in saprotrophic, pathogenic, and endophytic lifestyles between species with very similar genomes and alike gene reservoirs (Stenlid et al. 2017) or the spatio-temporal changes in functions relevant e.g. in proceeding wood decay (Zhang et al. 2016, 2019). In a few fungal species with established transformation systems, functional gene analyses were further assisted through molecular techniques, such as RNAi gene silencing in the ectomycorrhizal *Laccaria bicolor* (Kang et al. 2020; Pellegrin et al. 2019; Zhang et al. 2022), *Agrobacterium*-mediated insertional mutagenesis in the ECM fungus *Hebeloma cylindrosporum* (Doré et al. 2014), and by knocking-out genes in the white rot *Pleurotus ostreatus*, also in combination with transcriptome sequencing for comparison with wildtype transcriptomes (Wu et al. 2020, 2021).

RNA-seq of mixed transcriptomes (dual RNA-seq) from organismal interactions uncovers also responses by the host to the colonizing fungi. *Q. robur* for example reacts differentially on the distance to the three different ECM species *L. bicolor, Paxillus involutus* and *Pisolithus microcarpus,* but with a shared core transcriptional program of DEGs (differentially expressed genes) when in contact in roots colonized by either of the three symbiotic basidiomycetes. It suggests the presence of a common symbiosis pathway (CSP) in ectomycorrhiza in this oak (Bouffaud et al. 2020). In other work, long non-coding RNAs (lncRNAs) and non-coding microRNAs (miRNAs) of plant origin were detected by RNA-seq in *M. larici-populina*–infected poplar leaves, implicated in RNA-mediated mechanisms of host defense-related post-transcriptional gene regulation including *R* genes encoding plant immune receptors (Chen and Cao 2015; Li et al. 2016; Wang et al. 2017). Different poplar *R* genes providing resistance against rust were identified in the genome with the help of QTL mapping and by comparative transcriptomics of resistant and susceptible poplar genotypes. The QTL data and *R* allele structures are useful markers in resistant tree breeding (Nvsvrot et al. 2020; Wei et al. 2020). Most recently, cross-kingdom transfer of fungal miRNAs into host cells from the ECM fungus *P. microcarpus* to *Eucalyptus grandis* was evidenced by small RNA (sRNA)-seq and fluorescence in situ hybridization (FISH) assays and shown to facilitate symbiosis by silencing genes for immune receptors in the root cells (Wong-Bajracharya et al. 2022).

### Comparative genomics—between fungal species

Comparative studies of many annotated fungal genomes from different clades made it possible to better predict what constitutes the ecological role and lifestyle of a fungus. Thereby, characteristic gene gains, gene family expansions, and gene losses became evident and positive correlations of genes and functions to lifestyles can be found (e.g., Floudas et al. 2012, 2015; Gazis et al. 2016; Hage et al. 2021; Hage and Rosso 2021; Haridas et al. 2020; Kohler et al. 2015; Ruiz-Dueñas et al. 2021; Zanne et al. 2020). Accordingly, the significant presence and absence of particularly important gene families involved in the degradation of plant cell wall polymers are recorded in the CAZy database (http://www.cazy.org) of carbohydrate-active and auxiliary activity enzymes (for deeper insight into enzymatic fungal decay mechanisms and what is learned by comparative genomics see Kües et al. in prep.). Plant cell walls protected by lignin incorporation are especially difficult to degrade which became only efficiently possible when 295–300 million years ago basidiomycetous fungi acquired the genetic information for aggressive enzymes in lignin degradation (Floudas et al. 2012). For fungi living in mutualistic relationships with trees, such enzymes could be a functional burden for their particular lifestyles as plant cell walls need to remain functionally intact for plant cell survival, organismal contact, communication, and mutual nutrition (Balestrini and Bonfante 2014). Comparative genomics indeed revealed substantial losses of families of genes central to the enzymatic wood degradation system as recurring evolutionary adaptations in mycorrhizal species of different taxonomic lineages (Hess et al. 2018; Kohler et al. 2015; Looney et al. 2022; Martin et al. 2008, 2016; Miyauchi et al. 2020b; Romero-Olivares et al. 2021). Though, the loss of key cell wall decomposing enzymes from saprotrophic fungal ancestors may not be enough to enable symbiosis. In the genus *Amanita,* for instance, this loss is common between symbiotic and asymbiotic saprotrophic species. Mycorrhizal *Amanita* species however share ancestral genetic expansions enriched for regulatory functions and oxidative metabolism. These then diverged by later lineage-specific expansions of genes implicated in transport, sugar metabolism, terpenoid metabolism, and further oxidative functions probably employed in defense reactions (Hess et al. 2018). Changes in genome structure resulting from the loss of certain sets of genes responsible for the breakdown of plant cell walls, combined with lineage-specific expansions of other genes beneficial for establishing symbiosis, were also found in the evolution of *Russulaceae* and are considered fundamental processes in the development of ECM and its functional diversification within the ecological guild (Looney et al. 2018, 2022).

Despite the of loss of genes for key cell wall decomposing enzymes, there is still a functional fine-tuned attack of selective host cell wall polymers by specific enzymes in first host tissue colonization, which is in principle common with other fungal guilds of plant colonizing species, such as endophytes, biotrophic pathogens and hemi-biotrophic species in their growth phase with living plant cells before they enter their necrotrophic phase (Anasontzis et al. 2019; Bellincampi et al. 2014). Genome comparisons between different types of ascomycetous plant pathogens revealed that biotrophs have a lower number of genes for plant cell wall degrading than hemi-biotrophs and necrotrophs, as one possible adaptation to living in physiologically active plant tissues (Wang et al. 2022). Interactions with plant cell walls and plant cell wall degradation abilities of different fungal guilds are certainly the most important topic in the ecology of all kinds of tree-associated fungi. For reasons of space, this crucial matter is presented and discussed in more specific detail in the complementary article to this review paper (Kües et al. in prep.). In this paper, we concentrate predominantly on other genetic traits that determine functional lifestyles in terms of ecology and biodiversity of tree-associated fungi.

Comparative genomics for instance showed that pathogens may carry unique secondary metabolite (SM) biosynthesis gene clusters (BGCs) for toxins potentially effective on their specific plant hosts, such as on infected tissues of trees. Dutch elm disease (DED) *Ophiostoma* species possess for example a fujikurin-like BGC with a PKS (polyketide synthase) core gene, in contrast to the conifer sap-staining *Ophiostoma picea* and non-pathogenic members of the *Ophiostomataceae* family, but similar to some individual plant pathogens from other ascomycetous clades. The distribution independent of phylogeny suggests acquisition of the BGC by horizontal gene transfer (HGT) (Sbaraini et al. 2017). Likely also obtained by HGT, the poplar canker pathogen *Mycosphaerella populorum* expresses a chaetoglobosin-like BGC during growth on poplar wood (Dhillon et al. 2015). Wang et al. (2022) reported from comparative genomics a correlation with increasing numbers of gene SM clusters from biotrophic to hemi-biotrophic and necrotrophic pathogens. Typical for many ascomycetous plant pathogens, the North-American lethal laurel wilt *Raffaelea lauricola* and the *Eucalyptus* leaf blight pathogen *Calonectria pseudoreteaudii* have greatly increased numbers of distinctive BGCs as compared to the non-pathogenic *Raffaelea aguacate* and average levels generally found in ascomycetes (Ye et al. 2018; Zhang et al. 2020). On the other hand, the emerging beetle (*Pityophthorus juglandis*)-associated aggressive walnut pathogen *Geosmithia morbida* in the USA has a comparably small genome and, in contrast, less BGCs than non-pathogenic relatives. However, comparative bioinformatics evaluation of adaptive evolution in codon use (dN/dS = ratio of nucleotide non-synonymous substitutions per non-synomymous site/number of synonymous substitutions per synonymous site) revealed the low number of 38 genes with yet unclear functions being under positive selection in pathogenicity (Schuelke et al. 2017). Besides, increased numbers of BGCs can also be present in lines of ECM species. A significantly higher abundance of terpene SM gene clusters (23 ± 1.5) in the ECM genus *Suillus* has been connected as a functional option to host-fungal communication and probably host specificity (Lofgren et al. 2021), because volatile ECM-derived sesquiterpenes are associated with lateral root development in mycorrhization (Ditengou et al. 2015; Kües et al. 2018).

In the *Cryphonectriaeae* family, BGCs in *Cryphonectria* species are considerably lower in numbers than in different *Chrysoporthe* bark pathogens that affect *Eucalyptus* spp. and other *Myrtales*. BGCs with their core genes are much alike across pathogenic and non-pathogenic *Cryphonectria* sister species, indicating that the presence of BGCs alone makes the chestnut blight *C. parasitica* not yet an aggressive pathogen (Stauber et al. 2020). Coinciding, potential toxins of *C. parasitica* as much as tested (diaporthin, orthosporin, cryphonectric acid, anthraquinones) are not conclusively operative in fungal virulence against chestnut, or they may be toxins against other micobes (Lovat and Donnelly 2019). Comparative genome analyses of the ash-dieback pathogen *H. fraxineus* and its non-pathogenic sister species *Hymenoscyphus albidus* identified a single *H. fraxineus*–specific BGC, which is the hymenosetin biosynthesis gene cluster *hym* (Elfstrand et al. 2021). Hymenosetin is a 3-decalinoyltetramic acid that reacted antimicrobial against Gram-positive bacteria, filamentous fungi, and a few yeasts, but it was not phytotoxic in laboratory ash tissue bioassays, in contrast to the steroid viridiol (Cleary et al. 2014; Halecker et al. 2014) from a BGC (*vir*) conserved between the two fungi (Elfstrand et al. 2021). Viridol is produced by both species but at necrotic sub-effective concentrations (Junker et al. 2014). Comparative genome analyses detected between *H. fraxineus* and *H. albidus* strong genomic synteny and few genes with positive selection in *H. fraxineus*, mostly restricted to BGCs and putative vegetative incompatibility genes (*vic* genes, alternatively named *het* genes) for HET-homolog proteins mediating nonself allorecognition (heterokaryon incompatibility). Antibiosis as an adaptive advantage of specific SMs to combat other microbes in the fungal niches is discussed (Elfstrand et al. 2021).

### Comparative genomics—within fungal species

On smaller organismal scales within the same or closely related species, genomic comparisons on the whole sequence level and by deduced molecular markers can reveal important effects and consequences of reproduction modes on gene flow, fungal lifestyle specializations, host shifts, and co-evolution with hosts (Coetzee et al. 2020; Gladieux et al. 2014). Severe invasive pathogens with a change of host often spread clonally. This is documented e.g. with RAPD and PCR–RFLP markers for DEDs of North American and Eurasian origin (Brasier and Kirk 2010; Katanić et al. 2020), and with microsatellites for American and European lines of the chestnut blight fungus *C. parasitica*. The latter fungus originated initially from East Asia with native co-evolved tolerant chestnut species (e.g., Japanese *Castanea crenata* and Chinese *C. mollissima*) and has from there and from North America repeatedly been introduced into Europe as a severe disease on the European *C. sativa* (Demené et al. 2019; Dutech et al. 2010; Kubisiak et al. 2007; Lovat and Donnelly 2019; Milgroom et al. 2008).

Marker analyses can furthermore detect hybridization events across existing genetic incompatibility and mating barriers between species, subspecies, and clones, with possible relevance for virulence and selection pressure on sexual reproduction employing alternate mating types. Three invasive DED lineages (first *Ophiostoma ulmi* from Asia around 1900, then the Asian *O. novo-ulmi* subsp. *novo-ulmi* with a spread in Europe from East to West between 1940 to 1970, and afterwards subsp. *americana* from the mid-1950s to the 1970s from West to East) with permeable genetic barriers are documented in two devastating pandemics in Europe, with replacements of *O. ulmi* more lately by the more aggressive *O. novo-ulmi* subspecies and fundamental changes in genetic population structures within invaders (Brasier et al. 2021). Natural DNA introgressions from species *O. ulmi* to *O. novo-ulmi* and between *O. novo-ulmi* subspecies around pathogenicity and mating type (*MAT*) loci with impact on fitness have been demonstrated by whole genome analyses to be main drivers of genomic diversity (Et-Touil et al. 1999, 2019; Hessenauer et al. 2020). The alternate *MAT-1* allele was transferred in Europe from *O. ulmi* to *O. novo-ulmi* which existed first only as MAT-2 (Paoletti et al. 2006). By now, *MAT-1* allel frequencies in *O. novo-ulmi* subsp. *americana* populations in Europe increased up to 32 and 43% and *vic* loci introgressed from resident *O. ulmi* are now nearly randomly distributed in the sexually outcrossing hybrid populations (Brasier et al. 2021). Directional selection in the current post-epidemic phase in Europe is on higher growth rates and increased aggressiveness (Brasier et al. 2021; Brasier and Webber 2019).

As a second example, alternate mating type distributions and vegetative incompatibility tests together with SNPs revealed that the heterothallic ascomycete *H. fraxineus* reproduces sexually with high gene flow in its rapid spread across Europe, with degrees of genetic diversity on local spatial scales reflecting differences in the timing of arrival (Gross et al. 2012, 2014; McMullan et al. 2018; Nguyen et al. 2016a; Orton et al. 2018). A bimodal distribution of CEG (core eukaryotic gene) sequences into two haplotypes deduced from sequenced genomes support the foundation of the European pathogen population by only two genetically divergent individuals (McMullan et al. 2018). *H. fraxineus* is asymptomatic with significantly higher genetic diversity on *Fraxinus mandshurica* in its native Asian environment in Far East Russia and Japan (Cleary et al. 2016; McMullan et al. 2018; Zhao et al. 2012). By fecundant production of recombinant offspring under favorable environmental conditions, the two pathogenic haplotypes that invaded Western countries with a host change to *F. excelsior* can and did easily outcompete the native homothallic European ash endophyte *H. albidus* which is less-reproductive by the haploid selfing (Hietala et al. 2018). The native selfing species *H. albidus* is considered a probable evolutionary dead-end by comparably higher genomic DNA erosion levels, through less efficacious meiotic purifying selection of deleterious mutations and a significant influence of a genomic accumulation of transposable elements (TEs). The invasive *H. fraxineus* is even richer in TEs by an additional 13 Mb of genomic DNA but appears to be more effective in repeat-induced point mutations (RIP) as a fungal defence mechanism in inactivating multiplied TE families (Elfstrand et al. 2021). Regarding the further evolution of the sexually successful *H. fraxineus*, there is the paramount danger of unforeseen migration of further alleles of almost all genes of the species from the large Asian gene pools into Europe (Cleary et al. 2016; McMullan et al. 2018).

Whole genome sequencing of individuals of *C. parasitica* discovered signatures of restricted recent gene flow in main clonal lineages in France and one specific lineage with strong recombination, suggesting potential changes in the mode of reproduction from asexual to sexual (Demené et al. 2019). Allelic diversity of PCR-amplicons of *vic* loci and a low multilocus linkage disequilibrium suggested the same in Croatia (Mlinarec et al. 2018). Though, there can also be a fitness advantage of asexual reproduction for the expansion of highly adaptive lineages. The *MAT-1* allele dominates in southeastern European populations by the very aggressive and successful clonal S12 line of mainly North American genetic origin, with a frequent and ongoing admixture in European populations. Deep genotype sampling and sequencing revealed that the dominant S12 clone arose likely under loss of the *MAT-2* mating type as a highly adapted expansive secondary bridgehead invasion from a mixed mating type population in southern Switzerland, northern Italy, or the northern Balkans. Ongoing TE activities are seen in insertion polymorphisms as a high degree of genetic differentiation within the invasive European S12 lineages (Stauber et al. 2021). However, the overall TE content (ca. 10% of the genomes; mostly Gypsy family TEs) and RIP frequency in a North American *C. parasitica* reference strain and a native Japanese strain are comparable (Demené et al. 2022). Moreover, there is only poor association in this species between TEs and genes with possible functions in pathogenesis (Demené et al. 2022; Stauber et al. 2021). However, kingdom-wide inventories observed higher TE-insertions in genes of plant pathogens and of animal-related fungi than in species of other fungal lifestyles, implying that TE-interlinked rapidly evolving genomic compartments may shape key adaptations in pathogen evolution, e.g. by mediating possible accumulation in the regions of effector genes for host manipulation (Muszewska et al. 2019; Torres et al. 2020). Meanwhile, the example of *C. parasitica* shows that suchlike derived rules are not necessarily consistently uniform across species and situations, much like other genetic traits observed as more typical adaptations in particular lifestyles (for other prominent features see below). Regarding ECM, species of the *Russulaceae* for example are characterized by an expansion of genome size through increased TE content, dense aggregations of TEs, an association of genes for small secreted proteins (SSPs) with TE "nests", a reduction in SM gene clusters, and loss of genes for plant cell wall-degrading enzymes (PCWDEs). Some features are shared with the saprotrophic sister species *Gloeopeniophorella convolvens*, i.e., TE expansion, reduction in SM gene clusters, and loss of some PCWDEs (Looney et al. 2022). As mentioned already above, typical basidiomycete ECM species arose polyphyletically in evolution through corresponding gene losses from saprotrophic white rot fungi, which enzymatically can decay recalcitrant lignocellulosic wood (Floudas et al. 2012; Kohler et al. 2015; further reading in Kües et al. (in prep.)). Accelerated genome evolution using TEs, for example, can apparently prime an evolutionary switch in fungal lifestyles (Looney et al. 2022).

Comparative genomics of sufficient numbers of isolates allows experimental access to a breakdown of host resistances as a further target, even to critical single gene mutation events. To get insight into Rmlp7 resistance breakup in poplar, an initial GWAS thus considered the virulence of 76 re-sequenced isolates of *Melampsora* poplar leaf rust as phenotype and the genotypes as alleles and identified SNPs significantly associated with virulence. Several genome scans and selective sweep detection methods computed allele frequencies and pointed to a single rust avirulence candidate gene for a unique *Melampsora* SSP of 219 amino acids of unknown function. A non-synonymous substitution (G81S) in the effective allele overcame the poplar Rmlp7 resistance (Persoons et al. 2022).

### Small secreted proteins and their functions

An important step for all in planta-living fungi is the invasion into the host tissues, regardless of whether symbiotic, pathogenic, or endophytic. Particular attention in genome comparisons is thus paid to definitions of the fungal secretomes, i.e., the complete assemblies of expressed secreted proteins, with expected functions for infestation, such as host attack, fungal defense including detoxification of phytoalexins, communication with the specific hosts, and nutrition of the intruder (de Queiroz and Santana 2020; Zeilinger et al. 2015). Interesting genes of large families of often lineage-specific and rapidly evolving SSPs (< 300 amino acids, with an N-terminal secretion signal) were observed in genomes of ECM fungi localized in repeat-rich genome islands (de Freitas Pereira et al. 2018; Doré et al. 2017; Hess et al. 2018; Lofgren et al. 2021; Martin et al. 2008), as well as in genomes of endophytes (Knapp et al. 2018) and pathogens (Denton-Giles et al. 2020; Duplessis et al. 2011a; Muszewska et al. 2019).

RNAi gene silencing revealed the first mycorrhiza-induced SSPs (MiSSPs) to encode essential secreted effector proteins for the root mycorrhization process of *L. bicolor* and its interactive communication with the poplar hosts in their extracellular space (apoplast) or after translocation inside of host cells. LbMiSSP8 displays a C-terminal repetitive DW[K/R]R motif containing a KEX2 protease cleavage site and resulting peptides have a proposed function in hyphal aggregation in outer ECM mantle and apoplastic Hartig-net formation. LbMiSSP7 interacts with intracellular host Trihelix transcription factors in the regulation of jasmonic acid-signaling (Daguerre et al. 2020; Kang et al. 2020; Pellegrin et al. 2019; Plett et al. 2014). The *Pisolithus albus* PaMiSSP10b protein is secreted during root colonization and enters host cells. When expressed in transgenic *E. grandis*, it altered the host polyamine biosynthesis and prepared for mycorrhization via interaction with a host adenosyl-methionine decarboxylase (Plett et al. 2021).

From the arsenal of SSPs in *Melampsora* poplar leaf rusts, some candidate genes were cloned heterologously into herbal plants (*Arabidopsis thaliana*, *Nicotiana benthamiana*) and found to suppress plant immunity (Madina et al. 2020; Petre et al. 2015) and to reduce callose deposition at host plasmodesmata with an increase in plasmodesmatal fluxes (Rahman et al. 2021). Several cloned *SSP* genes from different *Heterobasidion* conifer root and stem rot pathogens induced necrotic responses in transient *N. benthamiana* transformants (Raffaello and Asiegbu 2017; Wen et al. 2019). Recombinantly produced *Ciborinia camelliae*-like SSPs (CCL-SSPs) from different necrotrophic *Sclerotiniaceae* induced necrosis in tested *Camellia* petals when coming from broad host range pathogens with only one *CCL-SSP* gene (i.e. *Botryotinia fuckeliana*, *Sclerotium sclerotium*). On the contrary, no or hardly any necrosis was seen for 10 other *CCL-SSP* genes that originated from the specialized *Camellia* flower pathogen *C. camelliae* which has a unique high number of a total of 73 *CCL-SSP* genes. KO mutants demonstrated that, despite functioning in the induction of necrosis, at least in *B. fuckeliana* the gene is not essential for full virulence (Denton-Giles et al. 2020).

Plant cell death was promoted in treatments of *Nicotiana tabacum* with a recombinantly produced *Heterobasidion* cerato-platanin (CP)–like SSP (Chen et al. 2015). A CP-like protein was one of only two highly expressed SSPs in the phloem of 2-year-old *F. excelsior* seedlings infested by the invasive *H. fraxineus*, by which it differed from the related native European ash endophyte *H. albidus* that expressed none (Stenlid et al. 2017). Considered pathogen-associated molecular patterns (PAMPs) or microbe-associated molecular patterns (MAMPs), the fungal-specific CP-like family of phytotoxic proteins is a stimulator of plant defense responses in colonization by pathogenic and beneficial fungi. Broadly distributed also in other guilds in the *Dikarya*, CP-like proteins can self-assemble into films on hydrophobic/hydrophilic interfaces, bind to chitin and *N*-acetylglucosamine oligosaccharides and exert also functions in fungal cell walls in growth and development (Gaderer et al. 2014; Luti et al. 2020), including fruiting body production (Almási et al. 2019; Krizsán et al. 2019).

Cysteine-rich hydrophobins as another common family of fungal-specific SSPs have highly diverged sequences and evolved in two distinct classes to cover aerial hydrophilic hyphal surfaces as an insoluble self-assembled amphipathic amyloid monolayer, rendering them hydrophobic. They control surface recognition and line air channels in complex tissues for gas exchange (Wessels 2000; Wösten and Wessels 1997). Encoded in lineage-increased gene families, specific gene copies were found differentially expressed in ECM species during the mycorrhization stage in the outer fungal mantle around and in the Hartig' net in plant roots, and in a preferred host more than in a less favored one (Plett et al. 2012; Rineau et al. 2017; Sammer et al. 2016). Hydrophobin genes exist also in species of other fungal guilds, often also in high gene numbers, but not always (Mgbeahuruike et al. 2013; Ohm et al. 2010; Plett et al. 2012; Stajich et al. 2010). Class I hydrophobins expressed in mycorrhizal interactions have significantly higher proportions in the N-terminus of exposed hydrophilic amino acids for interactions with fungal polysaccharides. This led to speculations about a stronger attachment of the amphiphilic proteins to the fungal polysaccharides in the mycorrhizal tissues compared to free-living mycelium (Rineau et al. 2017). In the chestnut blight *C. parasitica*, a class II hydrophobin named cryparin is required for the eruption of stromal pustules from under the host periderm for spore release while it is dispensable for host infection as shown by KO mutants (Kazmierczak et al. 2005; Lovat and Donnelly 2019; Table 2). Cryparin has a unique GS/T-rich N-terminus (36% hydrophilic amino acids) and is unusual in that it attaches to fungal hyphae in liquid presumably by lectin reactions (McCabe and Van Alfen 1999). Among changes in hundreds of other DEGs, cryparin expression is down-regulated by the single-stranded RNA Cryphonectria hypervirus 1 (CHV1) infection (Chun et al. 2020) which in viral disease reduces the pathogenic potential (hypovirulence), concomitantly with fungal yellow-orange pigmentation and sporulation in wounds of trees, by altering gene expression patterns of its fungal host. Therefore, CHV1 is used as a natural sustainable biocontrol agent to protect chestnuts (Eusebio-Cope et al. 2015; Rigling and Prospero 2018; Stauber et al. 2022).

**Table 2:** Catalog of virulence factors acting in pathogenicity of *Cryphonectria parasitica* on susceptible chestnuts

Gene	Function	Effects on virulence and growth characters by single gene knock-out or gene knock-down	Reference
*CpD14*	Strigolactone hydrolase	Suppression of fungal growth by analogs of strigolactone-type phytohormones under controlled growth conditions; no effect on host infection through wounds; suspected activity in epicormic bud outgrowths around the canker region of the bark lesion	Fiorilli et al. (2022)
*CdAoh1*	Oxalacetate acetylhydrolase	Reduction in pigment production; dramatic reduction in virulence with small cancers	Chen et al. (2010)
*CpLcc3*	Tannic acid-inducible laccase 3, a phenoloxidase	Reduction in growth on tannic acid; reduced virulence with smaller necrotic areas	Chung et al. (2008)
*CpKex2*	Kexin-like protease involved in protein secretion	Decrease in conidiation; reduction in virulence with smaller cancers; reduced browning in virulence apple tests	Jacob-Wilk et al. (2009)
*CpCrp*	Cryparin, a class II hydrophobin	Hydrophilic hyphae; no other alteration in growth phenotype; no alteration in virulence degree; required for eruption of stromatal pustules formed normally underneath the bark	Kazmierczak et al. (2005)
*CpSep1*	Septin (cytoskeletal small GTPase)	Retarded growth; hyperbranching; abnormal aerial mycelium; increased conidiation and change of conidia morphology; increase in sensitivity to oxidative stress; smaller necrotic areas with less stromatal pustules; reduced browning in virulence apple tests	Jo et al. (2019)
*CpG1*	Gα-subunit in G‐protein signaling	Reduced growth rate; reduced orange pigmentation; reduced conidiation; reduction in phenoloxidase activity; reduction of *CpCrp* expression	Gao and Nuss (1996); Segers et al. (2004)
*CpGb1*	Gβ-subunit in G‐protein signaling	Reduced pigmentation; reduced conidiation; reduced virulence	Kasahara and Nuss (1997); Kasahara et al. (2000)
*CpRgs1*	Regulator of G-protein signaling	Growth reduction; no pigmentation; no conidiation; complete loss of virulence; cellular accumulation of G-protein subunits; reduction of *CpCrp* expression	Segers et al. (2004)
*CpBdm1*	Phosphoducin-like protein regulating Gβ stability; target of protein kinase CK2 (casein kinase II) for phosphoactivation	Reduced pigmentation; reduced conidiation; reduced virulence	Kasahara et al. (2000); Salamon et al. (2010)
*CpSte11*	Pheromone-response MAPKKK (mitogen-activated protein kinase kinase kinase)	Reduction in size of necrotic lesions; reduction in number and size of stromatal pustules	Park et al. (2012)
*CpKk2*	Potential pheromone response MAPKK (mitogen-activated protein kinase kinase)	Growth reduction; absence of aerial hyphae; reduced pigmentation; increase in phenoloxidase activity; increase in sensitivity to oxidative stress; dramatic reduction of virulence with no cancer formation within 8 weeks	Moretti et al. (2014)
*CpMk2*	Fus3/Kss1-like MAPK (mitogen-activated protein kinase)	Growth reduction; reduction of aerial hyphae; no conidiation; unusual dark-brown pigmentation; reduced phenoloxidase production; reduced virulence with smaller necrotic areas	Choi et al. (2005)
*CpSte12*	Ste12 transcription factor homologue	Substantial reduction of virulence and female fertility; transcriptional regulation response similar to hypovirus infection	Deng et al. (2007)
*CpMk1*	Hog1-like MAPKK in osmotic stress regulation	Sensitivity to hyperosmotic stress; no pigmentation; reduction in conidiation; reduced levels of phenoloxidase expression; reduced virulence with smaller necrotic areas	Park et al. (2004)
*CpBck1*	Cell wall integrity (CWI) MAPKKK	Retarded growth with sporadic sectorization; thinner invasive hyphae; near-absence of aerial mycelium; no conidiation; diffuse pigmentation of finally dark brown color; hypertrophoid globose or bulbous cells; propagation of intracellular hyphae ("cell within-a-cell"); defects in cell wall integrity; reduced virulence with smaller necrotic area	Kim et al. (2016a)
*CpKk1*	CWI MAPKK	Growth reduction; thinner hyphae and absence of aerial hyphae; reduction of conidiation; no pigmentation; increase in oxidative stress; reduced cell wall resistance; dramatic reduction of virulence with no cancer formation within 8 weeks	Rostagno et al. (2010); Moretti et al. (2014)
*CpSlt2*	CWI MAPK	Reduced growth rate; sectoring of colonies; near absence of conidiation and aerial hyphae; propagation of intracellular hyphae; abnormal pale-greyish brown pigmentation; reduced phenoloxidase activity; hypersensitivity to cell wall-disturbing agents; increased sensitivity against reactive oxygen species (ROS); drastically reduced virulence with small necrotic lesions and with hardly any stromatal pustule formation; down-regulation of downstream components of the CWI pathway	So et al. (2017)
*CpSahh*	*S*-Adenosylhomocysteine hydrolase	Slower growth rate; fewer aerial hyphae; loss of orange pigment; significant reduction in virulence; reduction in transcription of *Cpcyp1*, *Cpste12*, and heterotrimeric G-protein genes; increase in transcription of key genes for the methylation pathway	Liao et al. (2012)
*CpDmt1*	DNA methyltransferase	Reduced colony growth with sectoring; increased phenoloxidase activity; sensitivity to heat shock; increased stroma formation in larger necrotic lesions	So et al. (2018); Ko et al. (2021)
*CpDmt2*	DNA methyltransferase	Reduced stroma formation and smaller necrotic lesions	So et al. (2018); Ko et al. (2022)
*CpProdh*	Proline dehydrogenase	Inability to use proline in conversion to glutamate; less pigment formation; suppression of conidiation; more salt-tolerant; reduction in virulence	Yao et al. (2013)
*CpP5Cdh*	Δ1-Pyrroline-5-carboxylate dehydrogenase	Inability to use proline in conversion to glutamate; increased pigmentation; more salt-tolerant; reduction in virulence	Yao et al. (2013)
*CpCyp1*	Cyclophilin A, peptidyl-prolyl *cis–trans* isomerase	Tolerance to drug cyclosporin A; significantly reduced virulence with small canker sizes; reduction in transcription of key components of the heterotrimeric G-protein signaling pathway (*CpMk2*, *CpSte12*, genes for Gα, Gβ and Gγ); increase in transcription of *CpMk1*	Chen et al. (2011)
*CpPrb1*	Serine protease, subtilisin-like protease	Reduced aerial hyphae; reduced virulence with smaller cancers; down-regulation of several genes in the heterotrimeric G-protein signaling pathway; increase in autophagic bodies	Shi et al. (2014)
*CpAtg4*	Autophagy regulating protease	Decrease in aerial hyphae and conidia production; increased sensitivity to H_2_O_2_ stress; reduction in virulence	Li et al. (2022)
*CpAtg8*	Ubiquitin-like protein	Reduced aerial hyphae; reduced conidiation; loss of pigmentation; reduction of virulence with small cancers	Shi et al. (2019)
*CpBir1*	Inhibitor of apoptosis protein that localizes to the nucleus and the cytoplasm	Reduced aerial mycelium; abnormal branching and swelling; no conidiation; increased pigmentation; increased H_2_O_2_ sensitivity; accumulation of ROS; drastically reduced virulence with small cancers and no stromatal pustules; loss of virus transfer	Gao et al. (2013)
*CpUbi4*	Polyubiquitin	Reduced growth rate; reduced aerial hyphae; drastically reduced conidiation; sensitivity to heat stress; lost ability to incite cancers	Chen et al. (2018a)
*CpHsp24*	Tannic acid-inducible heat shock protein, molecular chaperone for laccase 3 function	Reduction in growth rate; sensitivity to heat shock; decrease in necrotic lesion;	Baek et al. (2014); Chun et al. (2022)
*CpVma1*	Vacuolar H^+^-ATPase catalytic subunit A	Reduction in growth rate; increased sensitivity against alkaline pH and Ca^2+^; reduced virulence with very small cancers	Li et al. (2019)

As another notable example, a family of three SSPs (Ssp1, Ssp2, Ssp3) is induced by the toxic aryl-alcohol 5-hydroxymethylfurfural (HMF) in the saprotrophic white rot *P. ostreatus* as regulators of the ligninolytic enzymatic system (in particular of secreted versatile peroxidase, extracellular aryl-alcohol oxidases, and intracellular aryl-alcohol dehydrogenases; Kües et al. in prep.). In response to environmental cues, they define the transition of the trophophase (growth phase) to the idiophase (stationary phase with secondary metabolite production) with a change of fungal primary to secondary metabolism (Feldman et al. 2017, 2019). Related SSPs occur widely distributed in litter- and wood-decaying and in mutualistic fungi, irrespectively of affiliation to specific fungal guilds (Feldman et al. 2020). Thaumatin-like (TLP) proteins and cupredoxins are other frequently observed fungal SSPs, expressed e.g. by many *Polyporales* during wood decay (Hage et al. 2021). Individuals of these may have β-1,3-glucanase activity as deduced from N-terminal sequences of the white-rot *Irpex lacteus* JGI ID1593168 from the TLP-F subfamily (Grenier et al. 2000) with 68/80% identity/similarity to glucanase TLG1 of the cultivated wood-inhabiting mushroom *Lentinula edodes* (Sakamoto et al. 2006). Others possibly act in copper homeostasis functions in fungal cell wall remodeling (Almási et al. 2019; Hage et al. 2021), or may show strong Fe^2+^ binding with Fe^3+^-reducing and hydroxyl-radical producing activities, maybe for lignin degradation (glycoproteins Glp1 and Glp2 of the wood degrader *Phanerochaete chrysosporium* belonging to the TLP-P subfamily; Tanaka et al. 2007). TLPs exist and are expressed also in plants as widely distributed pathogenesis-related proteins (PR-5 family of defense proteins). In *Pinus sylvestris*, resistance-related jasmonate-induced expression of its antifungal TLPs helps thus against infections of the root and stem pathogen *Heterobasidion annosum* (Šņepste et al. 2018). A TLP gene was identified in a GWAS as one hub gene of importance for pine tree breeding (Ding et al. 2022b). The antifungal activity of *Picea* TLPs is based on glucanase activity on fungal hyphal cell walls (Liu et al. 2021, 2022).

According to the above, the presence of genes for SSPs per se does not indicate effector functions in interactions and communication between fungi and living plants (Kim et al. 2016b). Indeed, also saprotrophic wood decay species can have numerous species-unique SSP genes (Hage et al. 2021). An overall high number of SSP genes is also not pivotal for successful host interaction. The ECM fungus *Amanita polypyramis* had the lowest number of SSP genes (86) compared to others (217, 282) in the genus, while most symbiosis-induced SSPs in *Amanita muscaria* (15/19) had no homologs in asymbiotic species (Hess et al. 2018). The chestnut pathogen *C. parasitica* has significantly less SSP genes than related saprotrophic ascomycetes of the same genus. When entering additional features, the machine-learning classifier EffectorP 2.0 software however discriminated among the fungi in *C. parasitica* the highest number of SSPs which were predicted to function effector-like in planta (Stauber et al. 2020). Effector-like SSPs are those SSPs that are transferred from an organism into the apoplast, the cytoplasm, or the nucleus of a host and there provoke and manipulate reactions in the host, including the plant immune system. Some others act as host-specific toxins which are usually expressed by necrotrophs (Lo Presti et al. 2015; see above). Overall, pathogens mostly tend to have a higher number and more diverse candidate effector-like SSPs than symbionts, these more than saprotrophs, and biotrophic more than necrotrophic pathogens (Kim et al. 2016b; Sperschneider et al. 2018) but, possibly depending on relationships of selected species, there are also reports with observations to the opposite (Wang et al. 2022). A recent software update of the machine-learning classifier to EffectorP 3.0 now distinguishes between apoplastic and cytoplasmic effectors and predicts biotrophs to have higher numbers of cytoplasmic effectors (Sperschneider and Dodds 2022). Also of significance, a tree host possesses gene families for its own effector-like SSPs which in turn can enter and manipulate the growth and morphology of fungal hyphae, such as *P. trichocarpa* SSPs hyphal growth and morphology of the symbiont *L. bicolor* (Plett et al. 2017).

### Transitions from endophyte to pathogen

The evolution of endophytism and transitions between fungal lifestyles are so far not well understood but will be selectable functions under ecological conditions (Suzuki and Sasaki 2019). Like symbiotic and pathogenic lifestyles (e.g., see Hibbett and Matheny 2009; Kohler et al. 2015; Ruiz-Dueñas et al. 2021; Wang et al. 2022), the origin of fungal endophytes is polyphyletic. A phylogenetic analysis of 241 euascomycetes (*Pezizomycotina*) disclosed endophytes to be in a transient state to and from pathogenicity (Arnold et al. 2009). A concept has emerged of a functional fungal continuum saprotrophic-endophytic-pathogenic (“the endophytic continuum”) in which hosts behave neutral or mutualistic or are attacked, behaviors which are combinatorically determined by fungal species, host genetic background, and environmental conditions and changes (Kogel et al. 2006). Many times, borders in the fungal lifestyles are not strict. There are overlaps in functions between members of different fungal guilds and individual functional switching is promoted by the environment, supporting the idea of functional continuums, such as saprotroph-endophytic-pathogenic or, also, symbiotic-endophytic-pathogenic (Zanne et al. 2020). Derived from another phylogenetic study of 163 fungi, some *Mucoromycota* and many *Ascomycetes* and *Basidiomycetes*, endophytes and nectrotrophs within some clades appear to have evolved into the other lifestyle at equal rates of change. However, ancestral character mapping suggested also that pathogenic biotrophs evolved from endophytes without regression and appear fairly stable in lifestyle (Delaye et al. 2013), probably due to their typically strong host dependency founded on a previously marked reduction in CAZyme (carbohydrate-active enzyme) genes associated with plant cell wall degradation (Zanne et al. 2020; Zhao et al. 2013). According to one particular study, a hemi-biotrophic pathogen (*Harpophora oryzae*, indeed of rice) on the other hand developed into a growth-promoting beneficial endosymbiont via loss of hundreds of genes (929 recognized in total) and expansion of families of others related to transposons (and genome decay) and for complex regulatory machines beneficial to signaling and transport of organic and inorganic substances (Xu et al. 2014).

Individual genetic adaptations, fixed in lifestyles and activated as possible responses to the environment, will control the flexibility organisms have to change their lifestyle. Adaptations may be based on broader genome rearrangements or can be lineage-specific on a fine scale and may probably focus on changes in gene expressions (Haridas et al. 2020). Unambiguous genomic signatures for lifestyle transitions, and also for the residing pathogenic potential, can thus be difficult to infer from genome comparisons alone, especially in clades with frequent lifestyle transitions and in cases of fungal generalists (Franco et al. 2022; Hill et al. 2022). Comparing e.g. genome sizes and the toolboxes of functionally annotated genes may not help (per se) in larger sets of species to reliably differentiate between lifestyles. Machine learning however distinguished with > 95% accuracy between saprotrophic and pathogenic lifestyles in the filamentous ascomycete class of *Dothideomycetes*, through the identification of six clades of genes (mostly containing single-copy genes) without yet a functional annotation that was unique to genomes of saprotrophs (Haridas et al. 2020).

Nevertheless, saprotrophs in the *Dothideomycetes* have proportionally higher numbers of CAZymes than pathogens (Haridas et al. 2020). However, there appear to be no dramatic differences in gene profiles for CAZyme families between biotrophic, hemi-biotrophic, and necrotrophic pathogenic species in clades from the *Dothideomycetes* (Ohm et al. 2012). Another study suggested a similar share of conserved CAZyme functions in the secretomes between the distinct lifestyles in a broader selection of endophytes and pathogens (necrotrophic, hemibiotrophic, and biotrophic) coming mostly from the ascomycete class *Sordariomycetes* (de Queiroz and Santana 2020). Particularly, many species of the order *Xylariales* (*Sordariomycetes*) associate as endophytes, pathogens or wood-and litter-decay fungi with forest trees, with frequent lifestyle changes between closely related species. A recent genome comparison of 96 species revealed loss of PCWDE genes to be common in endophytic versus saprotrophic species of the *Hypoxylariceae* but not among endophytic, pathogenic, or saprotrophic species in the sister clade *Xylariaceae*. Endophytic *Hypoxylariceae* tend to be ecologically more specialized than endophytic *Xylariaceae*, which, as host and substrate generalists, offer greater saprotrophic potential. Both clades collected multiple BGSs of different origins in their individual genomes (on average 71.2). Especially the *Xylariaceae* accumulated many BGSs (frequently > 80 and even > 100) of hyperdiversity (28.2% BGSs were unique to a single taxon and 30–41% of BGSs were unique to single isolates within a species), often obtained via HGT. Whether the higher number of BGSs broadens the pathogenic host ranges of *Xylariaceae* compared to the specialized *Hypoxylariceae* remains to be shown (Franco et al. 2022).

As already described above, the behavior in *F. excelsior* and the gene content of the endophytic homothallic *H. albidus* and the pathogenic heterothallic *H. fraxineus* indicate subtle while crucial differences between an endophytic and a pathogenic lifestyle on European ash (Elfstrand et al. 2021; Stenlid et al. 2017). These two fungi live harmless and asymptomatic as endophytes during the vegetation period within their native hosts and show an increase in biomass in senescent host leaves not until autumnal leave fall, with a potential to then switch to necro- and saprotrophic growth phases (Hietala et al. 2022; Inoue et al. 2019). *H. fraxineus* however performs this switch in European ash earlier in the summer season, possibly inflicted by a high infection pressure forcing fungal sporulation and dispersal in nectrotropic lesions (Hietala et al. 2022). The unique BGS for hymenosetin antibiotic production in *H. fraxineus* (Elfstrand et al. 2021) may influence the success of colonization and sporulation via (reciprocal) antagonisms with native ash endophytes (Haňáčková et al. 2017; Hietala et al. 2022; Schlegel et al. 2016). Determination of whole epi- and endophyte communities in European ash leaves in the longer run may provide more insight into possible regulatory interventions on interactive organismal levels carried out in planta (Hietala et al. 2022) and how native endophytes may help to protect their hosts against pathogenic invaders (Agostinelli et al. 2021; Martínez-Arias et al. 2021).

The discipline of evolutionary epidemiology addresses the co-evolution between a pathogen and its host. Pathogens evolve quickly to take advantage of ecological or environmental changes including shifts in hosts (Thines 2019), such as showcased by *H. fraxineus* with the jump from Asian ash species onto European ash. About 10% of the *H. fraxineus* proteome is estimated to participate in interaction with its host *F. excelsior*, among them many secreted cytochrome P450 oxidases that may contribute to the attack of the host (McMullan et al. 2018). What protects in contrast the same host against the endophyte *H. albidus* with a very similar genome? This might have been molded on both sides by the co-evolution of endophyte and host. As a possible example of co-evolution, comparative studies on the resistant and susceptible chestnut host’s sides indicated expression of candidate *S* genes (*prm4* for callose synthase; *dmr6* for conversion of salicylic acid) in the European *C. castanea* susceptible to the introduced *C. parasitica* but not in the Asian tolerant *C. crenata* (Pavese et al. 2021).

### Other fungal virulence factors

Virulence, which is the pathogen’s quality to overcome defense reactions, harm its host and cause disease, is especially broadly analyzed in *C. parasitica*. Well-working gene deletion and silencing protocols via efficient fungal transformation exist, standard virulence assays on chestnut stem/bark cuttings and also on apples as a foreign host are established, and hypovirus test strains of different intensities for fungus-mycovirus interactions from the standard field isolate EP155 were developed, most notably the prototypic EP713 by transfection with CHV1-EP713 (see e.g., Churchill et al. 1990; Faruk et al. 2008; Jacob-Wilk et al. 2009; Jo et al. 2019; Lan et al. 2008; Li et al. 2019; Rostagno et al. 2010; other references in Table 2).

Under mycelial fan formation, the invasive necrotrophic *C. parasitica* infects host stems through wounds and grows intercellularly in the bark and cambium of susceptible chestnuts. Mycelial fans apply pressure to split host cells, even lignified host cells and developing wound periderm. Toxins like oxalic acid excreted at the mycelial forefront kill the host cells in advance (Lovat and Donnelly 2019; Rigling and Prospero 2018). In accordance, KO of the gene for producing oxalacetate acetylhydrolase (*CpOah*) reduced much the virulence of the pathogenic fungus (Chen et al. 2010; Table 2), similarly as the recombinant expression of *Oxo* in susceptible chestnut confers resistance in the host and was performed most recently under control a wounding- and pathogen-responsive promoter of gene *win3.12* (Carlson et al. 2022; Onwumelu et al. 2022; Polin et al. 2006; see above).

Comparative transcriptomic and proteomic analyses identified multiple candidate genes to act in virulence which are repressed in response to host-protective CHV1 infestation of the fungus or to added tannin as a major phytoanticipin against pathogens present in the bark of chestnuts (e.g., in Barakat et al. 2012; Chun et al. 2020; Kim et al. 2012; Wang et al. 2016). The application of genome-wide omics techniques on the raising number of targeted mutants further aids the identification of crucial virulence functions (Andika et al. 2019). Large sets of differentially regulated genes and genes suspected to act in activation of other genes and post-transcriptionally on encoded proteins were in the meantime deleted or in some cases down-regulated by genetic manipulation for functional tests in virulence (Table 2). Phenotypic descriptions (in part extended beyond the documentation in Table 2, e.g. on what is happening in relation to sexual reproduction) were sometimes backed up by double-KOs for the evaluation of hierarchical functions, also by gene overexpression experiments, or in some instances by the production of constitutively activated mutant genes (for respective details see in references given in Table 2). With regard to virulence, a complex picture emerges of different signaling pathways acting in coherent but also opposing directions, independent of or with parallel effects on growth traits, cell wall qualities, perceptions of different types of stress, and defense strategies against toxic plant metabolites and other host measures (Table 2). While variations in genotypes of pathogens, viruses, and chestnut hosts play a role in the severity of responses (Ježić et al. 2021; Krstin et al. 2017; Nuskern et al. 2021), and there may be no simple unifying rules recognizable from the mutant data (Table 2), understanding better the processes of fungal virulence could be useful to find potential novel targets and breeding strategies to combat the disease.

## Meta-omics

The compact overview above on functional ecological roles of different fungal guilds in view of tree growth and performance is complex, but it is by far not complete in its complexity (Nilsson et al. 2019; Zanne et al. 2020). Usually, in laboratory work, culturable fungi have been examined separately under artificial conditions in terms of their actions, most commonly for some more or less aggressive decomposing species which are appreciated as good models for investigating wood decay (e.g., Alfaro et al. 2020; Castaño et al. 2021; Eastwood et al. 2011; Fernandez-Fueyo et al. 2012; Floudas et al. 2012; Hori et al. 2014; Janusz et al. 2018; Kuuskeri et al. 2016; Levasseur et al. 2014; Martinez et al. 2004, 2009). Laboratory data, however, do not reflect throughout nature, with the ever-varying biotic and abiotic parameters, community interactions in promotion, exploitation, or competition, and resulting biodiversity changes (Fig. 1) that for a complete functional system picture and estimations of degrees of functional redundancies need all to be considered.

More recently, research on model species turned more into laboratory analyses for the effects of combined species, but again, typical studies of this type concern mostly well-growing competitors in wood rot with occasional evidence of additive activities as well (Hiscox et al. 2018; Presley et al. 2020; Sugano et al. 2021) or are sometimes used in the search for superior antagonists against pathogenic wood rot fungi (Wang et al. 2022; Wen et al. 2019, 2022). However, first omics studies become possible in laboratory species combinations to gain insights into the processes of interactions. In combat zones for example, fungi strongly modify their transcriptomes in order to express different defense strategies and possibly also to change their nutritional strategies by unilaterally exploiting the other organism (Presley et al. 2020) or by acting in mutual synergy in nutrient turnover (Sugano et al. 2021).

The priceless value of fungal cultures for understanding their biological processes and ecological significance must thus not be negated (Yasanthika et al. 2022), but culture-independent omics-methods open completely different dimensions on meta-levels on communities and community actions in nature. Defining whole community structures with meta-omics techniques brings about a better understanding of their ecological niches, underlying modes of operations, interactions and environmental interferences, temporal and spatial biodiversity changes, and eventual disintegration. Meta-omics DNA and RNA techniques capture entire communities with the full diversity of the many so far unknown taxons, including also all non-culturable and never seen species. At the current time, the main focus on fungal meta-omics is still on taxonomy with complete species coverage by meta-barcoding based on sequencing of isolated bulk DNA (Yasanthika et al. 2022) while studies on meta-transcriptomes are gradually emerging.

### Meta-barcoding

Meta-genomics with HTS is by now well established in definitions of whole microbial communities directly from the total isolated DNA of environmental samples (Nilsson et al. 2019), collected from individual ecological (micro)niches of relevance. In the context of trees, fungal DNA can originate from distinct soil horizons, plant litter, wooden substrates, subterranean rhizospheres, aerial phyllospheres, living and dead plant tissues, or also the microbial aerobiome (Fig. 1). Temporally, it can be collected at different seasons in the year. Ecological scales under consideration vary from micro-niches over local, regional, and national plots to geographic and climate roles. Specific PCR-amplification and sequencing of ITS sequences (ITS1-5.8S rRNA-ITS2 region) assist most often through (meta-)barcoding in large-scale identification and taxonomy of entire fungal communities, i.e. of all cultivable as well as non-cultivable known and unknown species, and it gives information on individual OTU (operational taxonomic unit, used as a placeholder for any non-identified species) abundances in the complete community of organisms. Alternatively, collectively sequenced genomes of the community obtained by meta-genomics can be scanned for sequenced marker barcodes (Nilsson et al. 2019; Stewart et al. 2018; Tedersoo et al. 2022). Meta-barcoding is increasingly employed for entire fungal community identification in biological niches of tree and forest relevance. Curated expert functional annotation tools FUNGuild, Fun^Fun^, and FungalTraits link molecular taxa barcodes automatically to fungal lifestyles and ecological functions (Nguyen et al. 2016b; Põlme et al. 2020; Zanne et al. 2020). The latest tool, FungalTraits, better considers switches in lifestyles and shifts in trophic modes. This is information of significance for instance for fungal communities in senescing leaves and needles of individual tree species (Tanunchai et al. 2023) and in the succession in wood decay (Lepinay et al. 2021), whereby organisms exist varied as endophytes, pathogens, or saprotrophs depending on the viridity status of the respective plant substrates or when foliar endophytes as priority colonizers enter saprotrophically other downed plant substrates.

All-inclusive meta-barcoding provides much better overall coverage of the species present in a habitat and can also provide information on the relative abundance of species and their likely contribution to community actions. With respect to dead wood decay in forest ecosystem functioning taken as an example, assessment of fungal decay communities is typically based on inventories in forests of sporocarps (Arnstadt et al. 2016; Heilmann-Clausen and Christensen 2004; Rieker et al. 2022; Uhl et al. 2022). Contrary to evidently naïve expectations, sporophores formed on deadwood turned however out to be poor predictors of the changing composition of entire fungal guilds captured by molecular barcoding and of the associated actual fungal decay activities in the wood over time (Müller et al. 2020a; Purahong et al. 2018; Rieker et al. 2022).

Significant interplays between saprotrophic and mycorrhizal fungi are emerging in meta-barcoding projects, with fungal guilds or individual species undergoing niche partitioning in soil types and horizons, influenced by parameters such as climate and season, region, pH and humidity, nutrients, predominances of generalists or specialists, and more (Awad et al. 2019; Chaithaisong et al. 2022; Godin et al. 2019; Khokon et al. 2021; Peršoh et al. 2018; Žifčáková et al. 2016). ECM compositions are proven drivers of pine, beech, spruce, and oak forest tree growth, with three-fold differences in the growth rates based on nitrogen-acquisition modes of ECM specialists (defined in the study by ITSs from nearly 40,000 individual ectomycorrhizae collected from 137 plots throughout Europe; van der Linde et al. 2018) in organic and inorganic soils with resulting lower and faster tree growth, respectively (Anthony et al. 2022).

Forest management measures must take into account the insights gained from such studies on compositions of tree-associated mycobiomes in order to locally and globally best conserve the biodiversity necessary for a functioning forest environment (Bowd et al. 2022; Goldmann et al. 2015; Tomao et al. 2020). As emerging from current community studies, the composition/richness diversity of fine and of coarse deadwood in forests in different ways can positively relate to the richness of wood-inhabiting fungi (Baldrian et al. 2016; Brabcová et al. 2022). Intensive forest management with the removal of types of deadwood and logging operations may negatively influence fungal density and diversity of wood-decayers (Tomao et al. 2020), while wood decay dynamics may inadvertently accelerate in the context of global climate warming even under the reduction of the wood decomposer guild (Chagnon et al. 2022) because, among, fungal species richness can negatively correlate with wood decay rates (Fukasawa and Matsukura 2021). Guilds of ECM species may likewise deteriorate under the influences of management, such as under reductions in canopy cover, basal area of stands, and tree species (Tomao et al. 2020). Increases in biodiversity of different fungal guilds (wood, soil, ECM) as disturbances by forest management are however also reported (Behnke-Borowczyk et al. 2021; Goldmann et al. 2015).

With respect to serious alterations in native organismal communities, particular attention may be paid to introduced and dangerous invasive neophytic trees with incalculable mycorrhiza, possibly co-introduced from other biographical regions, and any concomitant transmission of potential pathogenic neomycetes with risks for native tree species; or, indigenous fungi may act pathogenic on the non-native trees after unforeseen host jumps (Beenken 2017).

Many of the current studies on tree health in nature focus on emerging destructive pathogens. However, endemic pathogens in healthy forests have important control roles over the conservation of forest biodiversity (Fodor and Hâruța 2022). Indeed, some kinds of pathogens in the fungal communities may offer potential endemic solutions in severe pest and pathogen management as ecosystem services. With this idea in mind, epi- and endophytic leaf mycobiomes were established from ash in metagenomics for the identification of antagonistic fungi for potential biological control agents (BCAs) as barriers to *H. fraxineus* infection (Becker et al. 2020; Cross et al. 2017). The holarctic ash endophyte *Hypoxylon rubiginosum* producing the antifungal metabolite phomopsidin in co-culture with *H. fraxineus* was identified as one promising candidate for the development of a native BCA to inhibit the pathogen in planta (Becker et al. 2020; Halecker et al. 2020). Crucial for an application of a favorable native BCA is an understanding of both, the dynamics of the pathogen in the broader fungal community over the whole vegetation season as well as the behavior of the antagonist in the broader environment. In terms of ash dieback disease, starting with meiotic sporulation of *H. fraxineus* underneath European ash trees in early summer, incidences of *H. fraxineus* in the epi-, but not necessarily in the endophytic mycobiomes of diseased and symptomless leaves increased (Agan et al. 2020; Cross et al. 2017). Occurrence within symptomless leaves revealed then a biotrophic phase in the life cycle in which *H. fraxineus* exists in close contact with living penetrated host cells, prior to a devastating switch to necrotrophy (Mansfield et al. 2018). Leaf necrosis correlated with the acceleration of the ash-specific *H. fraxineus* in the mycobiomes, declines in the diversity of biotrophs, and increases in varied ubiquitous facultative pathogenic endophytic ascomycetes (Cross et al. 2017). Leaf mycobiomes of tree species co-inhabiting forests with European ash showed close compositional similarities to those of ash trees, confirming that many of the mycobiome species are non-specific and generalists (Agan et al. 2020; Agostinelli et al. 2021). In particular, abundances of the black yeast-like ubiquitous *Aureobasidium pullulans* as typical epi- and endophytic foliar ascomycetous generalist (Andrews et al. 2002) were found to increase in the mycobiomes of symptomatic ash trees (Agan et al. 2020). Among some other endophytic ascomycetes with antibiotic and mycoparasitic potential (Barta et al. 2022; Becker et al. 2020; Bilański and Kowalski 2022), the exceptionally stress-tolerant *A. pullulans* with a genome well equipped with a broad catalog of stress-tolerance genes (Gostinčar et al. 2014) is another candidate for developing operational BCA strategies against ash dieback disease due to its strong antagonistic activities against tree leaf pathogens (Agan et al. 2020; Pinto et al. 2018).

### Meta-transcriptomics

Meta-transcriptomics as a first approximation identifies physiological activities in ecological niches and thus provides biologically informative functional insights beyond pure accounting of species abundances. Technically more challenging by the need of isolating sensitive RNA for multi-organisms' RNA-seq from difficult environmental probes, by the high abundances (ca. 95%) of disturbing rRNA (potentially solved for eukaryotes by polyA enrichment of mRNA), and among, also, by a gigantic amount of yet unknown genome background data for best functional annotation, first reports are emerging where sequence similarities to established genetic data in databases, motifs in deduced protein products and also parallel meta-genome sequencing of total isolated DNA were made use of (Fonseca et al. 2022; Hesse et al. 2015; Liao et al. 2014; Schneider et al. 2021). Reconstruction of smaller genomes from environmental metagenomes (metagenome-assembled genomes = MAGs) from individual bacteria has started with suitable bioinformatic tools and pipelines (Zhou et al. 2022) but for the larger genomes of the many non-model fungi in nature, and for the better transcript annotations of today's possible meta-transcriptomics, this will all still take time. Supportive activity tests in environmental samples or their extracts are feasible for a few more persistent types of enzymes, for instance, laccases and peroxidases. Backing for actual physiological activities through meta-proteomics and metabolomics is desired (Sebastiana et al. 2021), but also still goes beyond the current technical feasibilities of routine (Baldrian 2019).

In methodological proof of concept, meta-barcoding of PCR-amplified ITS fragments has lately been complemented by shotgun-metagenomic sequencing and small RNA meta-transcriptomics in order to characterize the core mycobiome of the leaf phyllosperic mycobiome of selected Brazilian rubber trees from its native neotropical Amazonian habitat. Yeast-like basidiomycetes with potential for antifungal-compound production and non-invasive ectophytic fungal pathogens (sooty blotch, flyspeck) were discovered in the barcoding to dominate in the core mycobiome, while the shotgun meta-genomics with the higher sequencing depth and meta-transcriptomic revealed extra abundances of insect pathogens (*Ophiocordycipitaceae*) and anaerobic fungi (*Neocallimastigomycota*), and *Trichoderma* spp. was detected in meta-transcriptomics as one of the physiologically most active fungal genera besides *Fusarium* and *Hypomyces* (Fonseca et al. 2022). Another recent study demonstrated for meta-genomic ITS and meta-transcriptomic RNA data from matched samples of root- and needle-associated fungal communities of *P. abies* in comparative bioinformatic workflows the principle power for functional community insights (in the study nutrient-related) but also the current limitations by coverage in available databases, such as of definitions at lower taxonomic levels and available reference genomes (Schneider et al. 2021).

Meta-transcriptomics now helps to unravel critical shifts in fungal lifestyles, e.g. from endophytic (asymptomatic) to pathogenic (symptomatic) by changes in gene expression in latent pathogens of *P. contarta* needles, and identified simultaneous shifts in host cell transcription, from upregulated defense genes in healthy needles as a taming response to fungal recognition to strong activation of a broad variety of stress-response genes upon disease outbreak (Ata et al. 2022). Meta-transcriptomic analyses under long-term anthropogenic N deposition (over 16 years) into maple forest floors indicated significant shifts in fungal community expression of fungal CAZyme gene profiles under influence of N, along with increases in abundances of *Ascomycta* as compared to *Basidiomycota* (typically less prominent in abundance in forest soils than the ascomycetes; Chaithaisong et al. 2022), decreases in expression of ligninolytic genes, and reduced litter decay in moderately decomposed O horizons (Hesse et al. 2015). Shifts in hardwood forest ECM-species communities correlated with C and N cycling in N-fertilized forest soils, with enhanced relative C to N mining activities by ECM in rhizosphere soils and by AM-species in bulk soils (Carrara et al. 2021). Long-term soil warming in a boreal *Picea mariana* forest resulted in changed fungal soil communities with more stress-tolerant taxa and allocation of their carbon resources to fungal cell metabolic maintenance at expense of litter decomposition (Romero-Olivares et al. 2019). In another meta-transcriptomic study in an unmanaged temporal *P. abies* forest, activities of ECM-fungi in soil went significantly down in winter seasons, along with host photosynthetic production (Žifčáková et al. 2016). Finally, in a recent study performed for a better understanding of tree nutrition in a designed, roofed outdoor cosm with defined irrigation with beech saplings collected from a natural forest in their natural soil, mixed transcriptomes from mycorrhized beech roots short-time (48 h) after feeding of defined nitrogen sources showed unanticipated independent reactions between ECM fungi and the host to sudden anthropogenic high nitrate and high ammonium fluxes in forest soil. The fungi were unaffected by the changes in their gene expressions while the host was unexpectedly not shielded by the fungi and responded by uptake and upregulating nitrogen-specific metabolic and protective gene functions (Rivera Pérez et al. 2022).

## Conclusions and outlook

Various genetic and genomic methods can now be applied to forest tree species and their associated fungal communities. In this review, we have summarized these techniques and examples of how they can be used in forest conservation genetics, tree breeding, association genetics, and for the investigation of associated fungal communities their ecological functions, and effective and rapid diagnostics. Since it is not possible to include all aspects in such a review, we selected examples from different research fields to give an overview of the topic. Due to decreasing sequencing costs and the availability of reference genomes, whole genome re-sequencing is feasible for several tree species now. Where this is not possible, different methods for genome complexity reduction are valuable alternatives to obtain genome-wide marker sets and to get insights into the molecular basis of adaptive traits or to predict the fate of forest tree populations in times of climate change. For these studies, different molecular methods can also be combined (e.g., RNA-seq for SNP identification, genotyping of these SNPs in a larger set of individuals, and finally association analyses to detect significant SNPs for the trait of interest). Promising molecular techniques, such as genomic selection, will help to substantially reduce the time of breeding cycles because decision-making is possible in the young tree age.

Bahram and Netherway (2022) recently reviewed the manifold mediator roles of fungi linking to organisms including trees and ecosystems as central ecological agents. Due to their smaller genomes compared to tree species, fungal genomes are easier to analyze. For instance, genome comparisons, which have only recently been started in forest tree species (e.g., to determine species barriers despite hybridization), are routinely conducted in fungal model species and led to the identification of the presence or absence of specific genes and expansions in certain gene families that are characteristic for different ecological fungal guilds. A deep understanding for instance of pathogen biology in its wide array of physiological and molecular determinants, host–pathogen interactions, and resistance mechanisms is fundamental for improved tree breeding programs. New genome-editing techniques may become more widespread as valuable tools in forest genetics research to better understand the gene functions of trees and their microbiomes and to confirm candidate genes identified by conventional association analyses.

Nevertheless, despite similarities in genetic backgrounds, there are no overall unifying rules explaining all possible situations of seemingly the same kind in nature. Studies of individual fungi are as important as defining whole tree-related communities, their collective ecological functions, and holobionts co-diversification. The composition of fungal communities can be influenced by various factors, such as forest management or changes in environmental conditions. Therefore, knowledge of balanced compositions of fungal communities and their functions should be considered in forest management. “The social life of trees and forests” of which fungi are a part is not to be neglected.

## Data Availability

Not applicable.
